# Electron-Microscopy-Based Epitope Mapping Defines Specificities of Polyclonal Antibodies Elicited during HIV-1 BG505 Envelope Trimer Immunization

**DOI:** 10.1016/j.immuni.2018.07.009

**Published:** 2018-08-21

**Authors:** Matteo Bianchi, Hannah L. Turner, Bartek Nogal, Christopher A. Cottrell, David Oyen, Matthias Pauthner, Raiza Bastidas, Rebecca Nedellec, Laura E. McCoy, Ian A. Wilson, Dennis R. Burton, Andrew B. Ward, Lars Hangartner

**Affiliations:** 1 Department of Immunology and Microbiology, The Scripps Research Institute, La Jolla, CA 92037, USA; 2 Department of Integrative Structural and Computational Biology, The Scripps Research Institute, La Jolla, CA 92037, USA; 3 Division of Infection and Immunity, University College London, London WC1E 6BT, UK; 4 International AIDS Vaccine Initiative, Neutralizing Antibody Center, The Scripps Research Institute, La Jolla, CA 92037, USA; 5 Center for HIV/AIDS Vaccine Immunology and Immunogen Discovery, The Scripps Research Institute, La Jolla, CA 92037, USA; 6 Skaggs Institute for Chemical Biology, The Scripps Research Institute, La Jolla, CA 92037, USA; 7 Ragon Institute of Massachusetts General Hospital, Massachusetts Institute of Technology and Harvard University, Cambridge, MA 02139, USA

**Keywords:** polyclonal antibodies, antibodies, antibody epitope mapping, electron microscopy, negative-stain EM, cryo-EM, vaccine, BG505, HIV, Env, SOSIP

## Abstract

Characterizing polyclonal antibody responses via currently available methods is inherently complex and difficult. Mapping epitopes in an immune response is typically incomplete, which creates a barrier to fully understanding the humoral response to antigens and hinders rational vaccine design efforts. Here, we describe a method of characterizing polyclonal responses by using electron microscopy, and we applied this method to the immunization of rabbits with an HIV-1 envelope glycoprotein vaccine candidate, BG505 SOSIP.664. We detected known epitopes within the polyclonal sera and revealed how antibody responses evolved during the prime-boosting strategy to ultimately result in a neutralizing antibody response. We uncovered previously unidentified epitopes, including an epitope proximal to one recognized by human broadly neutralizing antibodies as well as potentially distracting non-neutralizing epitopes. Our method provides an efficient and semiquantitative map of epitopes that are targeted in a polyclonal antibody response and should be of widespread utility in vaccine and infection studies.

## Highlights

• Mapped polyclonal epitopes of HIV-Env-immunized rabbits via electron microscopy
• Followed development and specificity of neutralizing antibodies over time
• Identified a class of antibodies binding between the HIV Env trimer blades
• Neutralizing antibody class displays highly convergent structural features

## Graphical Abstract

**Figure:**
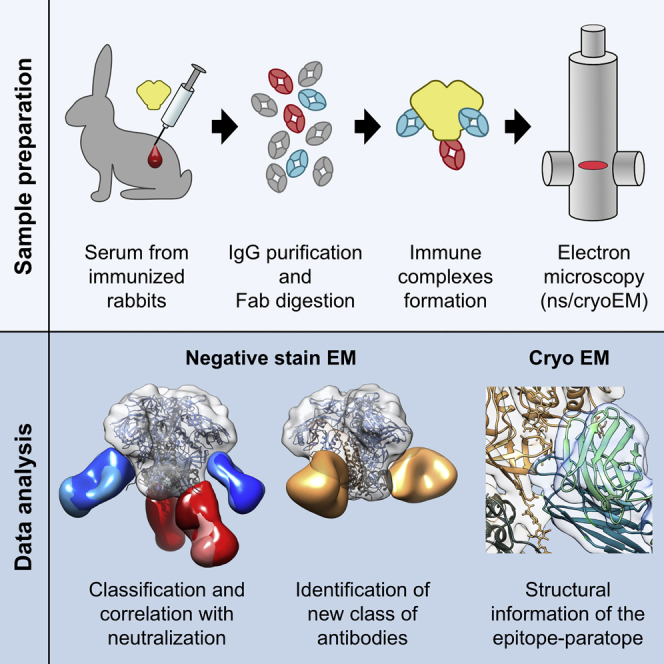

## Teaser

Comprehensive epitope mapping is fundamental to understanding immune responses. Bianchi et al. applied electron microscopy to study polyclonal antibody responses in HIV-Env-immunized rabbits. Different antibody specificities were identified and followed over time, revealing the source of neutralization, a distinct class of antibodies, and a highly convergent structural evolution of the neutralizing antibodies.

## Introduction

Classically, vaccines are composed of killed or attenuated pathogens or protein subunits derived from the pathogen surface. Although most successful vaccines are based on these approaches, highly antigenically variable pathogens, such as HIV, and pathogens that circulate in the population as a large number of serotypes have proven less tractable. A different approach based on isolating functional antibodies to the pathogen by studying their interaction with their targets and then designing vaccine candidates has been described (Burton, 2002, Burton, 2017, McLellan et al., 2013, Rappuoli and De Gregorio, 2016, Walker et al., 2009). For highly antigenically variable pathogens, broadly neutralizing antibodies (bnAbs), i.e., antibodies that can recognize multiple antigenic variants thereof, can usually be isolated only from a small subset of infected patients (McCoy and McKnight, 2017). The target for HIV bnAbs is the metastable envelope (Env) antigen, which consists of the two glycoproteins gp120 and gp41 arranged in a (gp120)_3_(gp41)_3_ trimeric assembly and sits on the surface of the viral particle. Stabilization is required for the generation of a recombinant molecule (SOSIP) that mimics the native trimer on the virus, and these recombinant trimers bind bnAbs and are antigenically native (Binley et al., 2000, Sanders et al., 2002, Sanders et al., 2013). Although some inferred germline versions of bnAbs are able to recognize the native Env trimer (Andrabi et al., 2015), the inferred germline versions of many other bnAbs typically fail to recognize both the recombinant trimers and the corresponding Env glycoprotein on the virus. However, engineered proteins have been designed to stimulate the precursor B cells of bnAbs (Briney et al., 2016, Escolano et al., 2016, Jardine et al., 2015, Medina-Ramírez et al., 2017, Sok et al., 2016, Steichen et al., 2016) and help advance structure-guided vaccine development against HIV according to the use of sequential immunogens (Escolano et al., 2016).

Although the first immunization experiments using native recombinant Env trimers (de Taeye et al., 2015, Pauthner et al., 2017, Sanders et al., 2015, Sok et al., 2017, Torrents de la Peña et al., 2017, Voss et al., 2017) and germline-targeting immunogens (Dosenovic et al., 2015, Jardine et al., 2015, Jardine et al., 2016, Sok et al., 2016, Steichen et al., 2016) in diverse animal models looked promising and were able to elicit tier 2 autologous neutralizing antibodies (nAbs) (Escolano et al., 2016), one of the rate-limiting steps in the iterative vaccine-development approach is in the analysis of the polyclonal immune response elicited by immunization. Serum neutralization assays and enzyme-linked immunosorbent assays (ELISAs) are typically used as relatively rapid readouts of the epitopes recognized by elicited antibodies but are restricted to previously characterized epitopes. Gaining a more detailed picture requires the generation of monoclonal antibodies (mAbs) (Escolano et al., 2017, McCoy and Burton, 2017, McCoy et al., 2016, Sok et al., 2017) and determination of their structures in complex with immunogens. This process is time consuming and limited to a relatively small number of samples. Such analyses typically focus on antibodies with a biological function (e.g., neutralization) and often leave the remainder of the humoral immune response less well investigated. Given the high cost and labor involved, unsuccessful outcomes of vaccination experiments are hardly ever analyzed in detail, and the reasons that a given immunogen might fail to generate a neutralizing response can remain unknown. More recent efforts to use deep sequencing of the B cell receptor (BCR) repertoire of responding B cells have considerable limitations because, in most cases, the heavy-light-chain pairing is lost (as reviewed in McCoy and Burton, 2017). Next-generation sequencing (NGS) analyses can be used to study responses but require a considerable amount of pre-existing knowledge to interpret the sequencing data; unless characteristic features of bnAb sequences are known or a comprehensive reference database of previously isolated and sequenced pathogen-specific B cell clones or mAbs is available, these analyses rely on identifying changes between the frequencies of V region clonotypes or families and those of the pre-immune state. Approaches that couple NGS data with tandem mass spectrometry (MS/MS) analyses of affinity-purified antibodies (Boutz et al., 2014, Lavinder et al., 2014, Reddy et al., 2010, Wine et al., 2013, Wine et al., 2015) have enabled the identification of BCR sequences that are antigen specific without prior knowledge of their genetic signatures. These approaches have brought more comprehensive insights into B cell responses; however, they cannot provide direct information about the epitope recognized unless the sequenced BCR sequences are synthesized and expressed as antibodies for validation and gaining insight into their specificity.

Immunization of rabbits with HIV trimer BG505 SOSIP.664 has previously been used for determining the immunogenicity of recombinant, native-like Env trimers (de Taeye et al., 2015, Klasse et al., 2016, McCoy et al., 2016, Sanders et al., 2015). These prior studies employed intramuscular prime immunization with 30 μg of BG505 SOSIP.664 with Iscomatrix adjuvant at day 0 and then booster immunization using the same formulation at weeks 4 and 24. BG505 SOSIP.664 immunization was found to induce autologous tier 2 nAb titers. Neutralizing mAbs 10A, 11A, and 11B isolated from BG505-SOSIP.664-immunized rabbits led to the definition of a highly immunogenic glycan hole (GH) present on the surface of the BG505 Env in the vicinity of S241 (Klasse et al., 2016, Klasse et al., 2018, McCoy et al., 2016). Antibodies specific to this particular epitope were identified as the primary source of neutralization. Negative-stain electron microscopy (nsEM) illustrated that the antibodies approach the Env surface from the membrane-proximal side of the trimer (McCoy et al., 2016). Besides this class of tier 2 nAb, two tier 1 nAbs were identified—10B, which recognized the V3 loop, and 10C, which competed with CD4-binding-site bnAbs on gp120—but both bound poorly to Env trimers. Lastly, 12A-like antibodies that displayed weak autologous neutralizing activity but bound to a different epitope than 10A, 11A, and 11B were identified. 12A-mediated neutralization was impaired in the presence of a glycan at position N611. nsEM revealed that mAb 12A bound to an epitope in the vicinity of the PGT151 epitope but at a more canonical bnAb angle of approach than that of 10A, 11A and 11B. These studies offer information about the diversity present in the humoral immune responses, including non-neutralizing epitopes that could potentially be a distraction from eliciting nAb responses. Here, we describe a complementary approach that, compared with isolation of mAbs, elucidates a more complex landscape of the antibody response within polyclonal sera, including the genesis and evolution of antibody responses targeting different epitopes on Env. These data can be rapidly generated, i.e., within a week of blood collection, and help inform the iterative, structure-based vaccine design process.

## Results

### Immunization with BG505 SOSIP.664 Elicited Different nAb Titers in Rabbits

**Figure 1:**
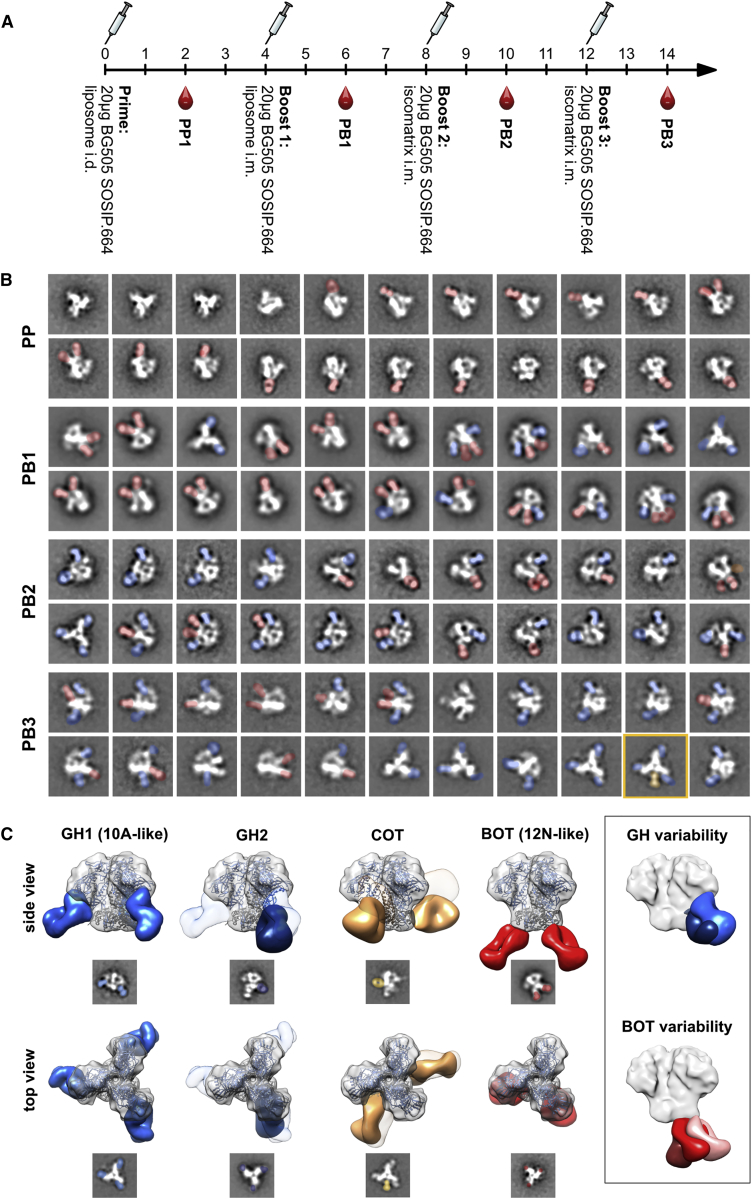
Epitope Mapping of the Antibody Response to BG505 SOSIP.664 Trimers in Rabbits by nsEM Defines Different Antibody Classes (A) Immunization schedule used for the four rabbits analyzed in this study. (B) Representative reference-free 2D class averages obtained after each BG505 SOSIP.664 immunization of rabbit 3417. Fabs bound to BG505 SOSIP.664 Env trimers (white) are highlighted with false coloring: red, bottom of trimer (BOT); blue, glycan hole (GH); orange, cleft of trimer (COT). (C) 3D reconstructions of the four basic antibody classes elicited by BG505 SOSIP.664 immunization of rabbits. Refined 3D models were fitted onto a low-pass filtered Env trimer reference structure (PDB: 5I8H; displayed as ribbons with gp120 in bright blue and gp41 in dark gray). For display of the surface, the density map for fully glycosylated BG505 SOSIP.664 (EMD: 5782) was also fitted with Chimera and displayed in semitransparent gray. Side and top views are shown for representative 3D reconstructions and 2D class averages. Densities for mAbs 10A and PGT151 are added as semitransparent references in the GH2 and COT 3D reconstructions, respectively. BOT-, GH-, and COT-specific antibodies are highlighted with red, blue, and orange, respectively. Representative 2D class averages are shown below each view. A comparison of the two GH classes and variations of the BOT epitope recognition is depicted in a box on the right. See also Figures S1 and S2.

For the purposes of the current investigation, we analyzed sera from four BG505-SOSIP.664-immunized rabbits (3417, 3418, 3419, and 3420) that had been extensively characterized in a previous study (McCoy et al., 2016). Rabbits were immunized four times with BG505 SOSIP.664 and bled 2 weeks after each immunization (Figure 1A). Sera obtained are referred to as PI, PP, PB1, PB2, and PB3 for pre-immunization, post-prime, post-boost 1, post-boost 2, and post-boost 3, respectively. Sera characterization using ELISA (Figures S1A and S1E) and neutralization assays (Figure S1B; McCoy et al., 2016) demonstrated that the antibody responses were comparable to those previously published (de Taeye et al., 2015, Klasse et al., 2016, Sanders et al., 2015). As in previous studies, only low titers of binding antibodies were induced after the prime (Figures S1A and S1E). However, the first booster immunization drastically increased these binding titers to their nearly maximum levels, such that subsequent immunizations afforded little further improvement. No autologous nAbs were induced by the priming immunization, and neither rabbit 3418 nor 3419 developed nAb titers above the detection level during the entire course of the immunizations (Figure S1B; McCoy et al., 2016). In the other two rabbits after the first boost, neutralizing titers substantially increased in rabbit 3417, but not in rabbit 3420, where neutralizing titers were observed only after the second boost. The third boost did not improve the already high nAb titers in rabbit 3417 and even resulted in slightly lower titers than observed in PB2 (Figure S1B). In the slower responding rabbit 3420, the third boost improved its neutralizing titers to around half the titer observed in rabbit 3417 (i.e., IC_50_ 63 versus 32 μg/mL). These data demonstrate that each animal responded somewhat differently in terms of nAb titers to the BG505 SOSIP.664 candidate vaccine.

### Biochemical Characterization of Polyclonal Antibody Serum and Generated Fabs

To determine the epitopes of the elicited antibodies without generation of mAbs, we devised a strategy to directly image immune complexes formed between the immunogen (BG505 SOSIP.664) and the induced serum antibodies by nsEM. Serum immunoglobulin G (IgG) was purified with a mixture of protein A and G affinity matrix and processed into fragments antigen binding (Fabs) with immobilized papain to prevent antigen crosslinking and aggregation due to the bivalent nature of IgG. Before nsEM, Fabs were subjected to biochemical quality control: purity and the correct size of the Fabs were confirmed by SDS-PAGE and size-exclusion chromatography (SEC) (Figures S1G and S1H). To investigate the effect of the IgG digestion protocol on the biological activity of antibodies, we determined neutralizing titers before and after IgG digestion. As depicted in Figure S1C, a considerable reduction of neutralizing activity was observed for the polyclonal serum when it was digested into Fabs. However, given that mAbs 10A and 11A also displayed a comparable loss in neutralizing activity when they were recombinantly expressed as Fabs, we concluded that proteolytic digestion did not have gross detrimental effects. Thus, we derived a method to reliably digest serum IgG into Fabs for further analysis.

### Polyclonal Image Analysis of Env-Fab Complexes by nsEM Reveals Different Classes of Antibodies

Given the limited amount of serum available and the low titers after the prime vaccination (Figures S1A and S1E), post-prime complexes could be identified only for rabbit 3417. Complexes were purified via SEC and deposited onto electron microscopy (EM) grids and imaged. For each sample, we collected 10,000–50,000 individual particle images that were submitted to reference-free 2D classification (Figure 1B). After priming, the early antibody response was completely dominated by bottom of trimer (BOT)-binding antibodies. These antibodies bound to a neo-epitope that is unique to the soluble Env trimer and does not exist on membrane-embedded Env. Hence, for the most part, these corresponded to non-neutralizing responses. After the first booster immunization, antibodies to the GH epitope (McCoy et al., 2016) were also identified in rabbits that developed neutralizing titers (3417 and 3420), but not in rabbits that did not mount neutralizing titers (3418 and 3419).

We performed 3D refinement that yielded 3D classes representing the most predominant immune complexes for all rabbits. When all reconstructions were overlaid and compared with prototypic mAbs, one BOT, one cleft-of-trimer (COT), and two GH-specific binding classes could be defined (Figure 1C). BOT antibodies recognized an epitope similar to the previously described bottom-binding mAb 12N (Kulp et al., 2017, McCoy et al., 2016) and were therefore binned into one class, although some variation in epitope and angle of approach was detectable in the 3D reconstructions (Figure 1C, box). Glycan hole 1 (GH1) class antibodies almost perfectly overlapped 10A, a prototypic GH-specific neutralizing mAb described earlier (McCoy et al., 2016). Relative to 10A, the second GH-specific class, GH2, bound in an orientation rotated approximately 90° along its longitudinal axis to the same region. COT class antibodies, i.e., antibodies that bound between the trimer blades, were found only in less than 1% of 2D class averages from rabbits’ 3417 and 3420 bleeds after PB1 (Figure S2A) and not in the 3D classes, indicating that these antibodies were not very abundant in comparison with the others. COT class antibodies were found to bind a membrane-proximal region located just below fusion-peptide-specific antibodies, such as PGT151. This region, typically referred to as the gp120-gp41 interface, is actually a cluster of overlapping epitopes that include COT, PGT151, and the previously described rabbit mAb 12A, which preferentially neutralizes viruses lacking the glycan at N611 (Figure S2B).

We hypothesized that the relative dominance of the BOT and GH antibodies prevented detection of COT antibodies in the 3D classes. Hence, our original BG505 SOSIP.664 probe was not sensitive enough to completely characterize the polyclonal response. When complexes were instead formed with Env trimers in which either the GH alone (Klasse et al., 2016, McCoy et al., 2016) or the GH and the bottom were modified to diminish antibody binding (Kulp et al., 2017), the frequency of detection of COT antibodies increased considerably in 2D class averages (Figure S2C), and 3D models could be generated from such immune complexes (Figures 1C and S2B). In contrast to human bnAb PGT151, which binds with a stoichiometry of 2 Fabs per trimer (Blattner et al., 2014), COT class antibodies bound with up to three molecules per trimer (Figure S2C, box). GH- and COT-specific antibodies could be concomitantly bound to the same cleft of the trimer (Figure 1B, box), indicating that there was no direct steric hindrance between these two classes.

**Figure 2:**
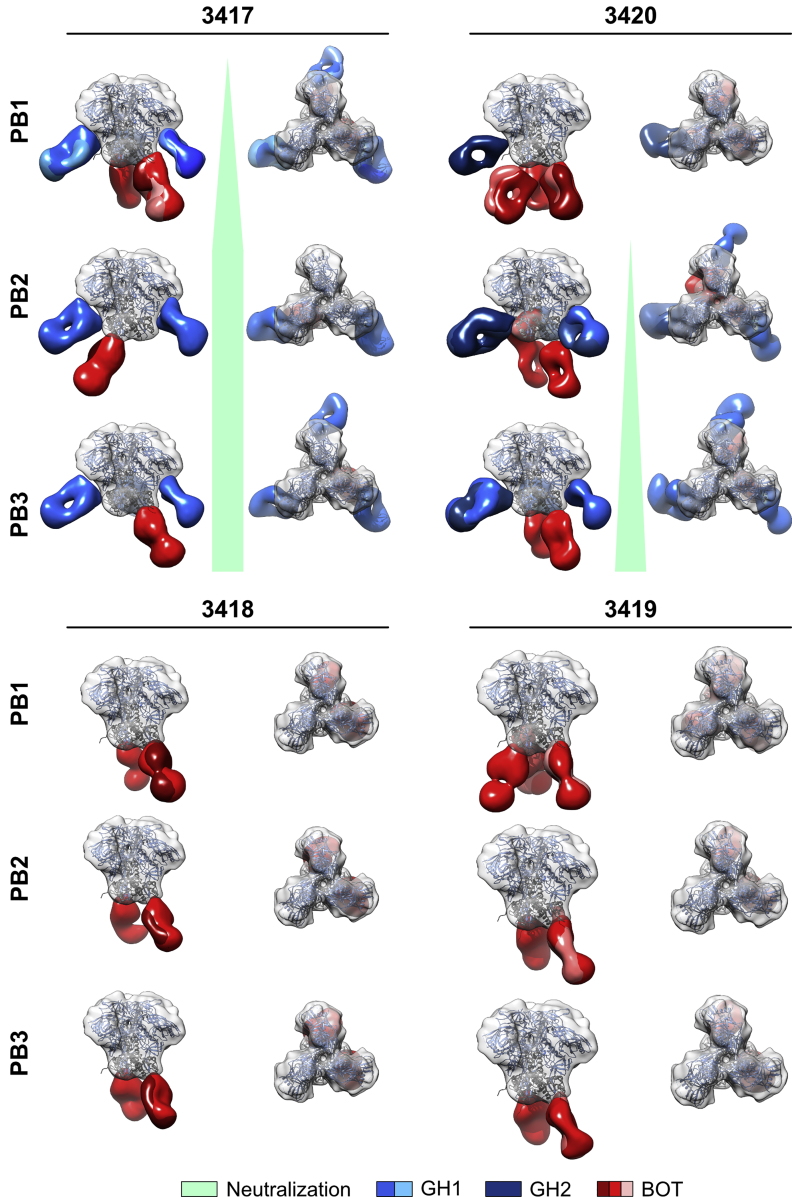
Epitope Mapping of the Antibody Responses in the Four Rabbits at Different Time Points during the Immunization Schedule Reveals that Neutralization Correlates with Appearance of GH1 Class Antibodies Refined 3D models were fitted onto a low-pass filtered Env trimer reference structure (PDB: 5I8H; displayed as ribbons with gp120 in bright blue and gp41 in dark gray). Densities corresponding to Fabs were separated and colored. For display of the surface, the density map for fully glycosylated BG505 SOSIP.664 (EMD: 5782) was aligned as described above and rendered in semitransparent gray. Side and top views are displayed. The bright-green wedge (between the side and top views) illustrates the development of autologous neutralizing titers, correlating with GH1 class antibodies, in two of the rabbits at different time points. See also Figure S1.

BOT-specific antibodies were the first and only class of antibodies detectable after priming (Figure 1B) and remained detectable throughout the course of immunization in all rabbits (Figure 2). In the two rabbits that developed autologous neutralizing titers (3417 and 3420), the differences in the kinetics of the development of neutralizing titers was also reflected in the classes of antibodies found. In the rapidly responding rabbit 3417, the appearance of GH1 antibodies coincided with the development of neutralizing titers at PB1, and GH1 remained the only GH-binding class of antibodies. In contrast, in the slowly responding rabbit 3420, GH2 but not GH1 class antibodies were detectable at PB1 (Figure 2). The fact that no neutralizing activity was found in this rabbit at PB1 (Figure S1B) suggests that the GH2 class of antibodies did not confer substantial neutralization activity against BG505. However, when autologous neutralizing activity became detectable in rabbit 3420 after PB2, GH1 antibodies became readily identifiable in the 3D reconstructions (Figure 2). These data suggest that GH1 class antibodies, like mAb 10A, were predominantly responsible for the neutralizing activity in these BG505-SOSIP.664-immunized rabbits.

**Figure 3:**
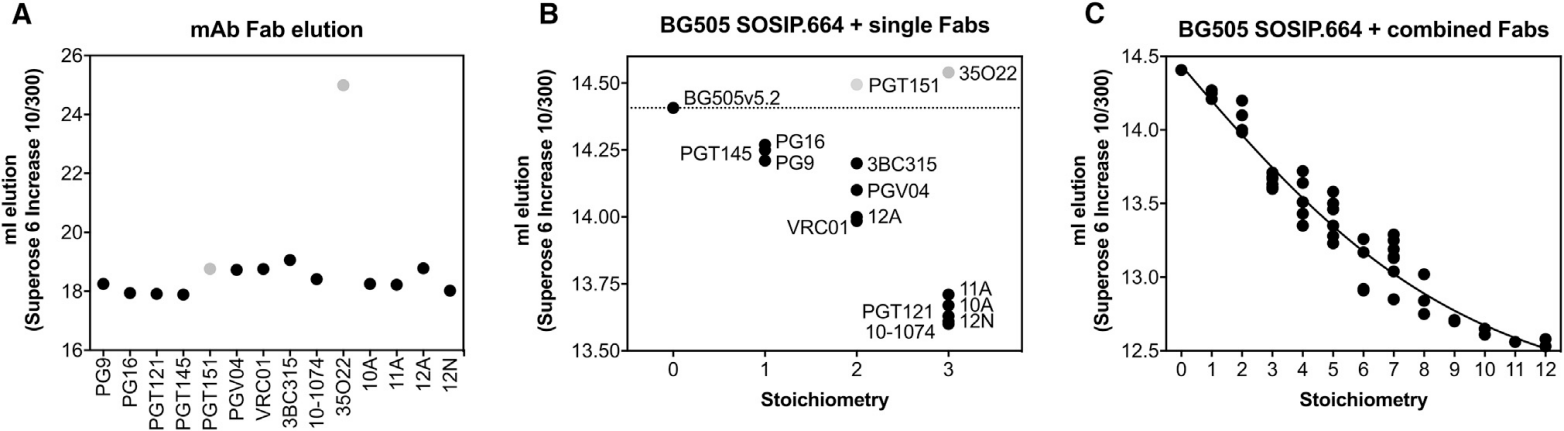
Generation of a Standard Curve for Measuring Fab Occupancy in Immune Complexes (A) Elution volumes of Fabs purified from the indicated mAbs. (B and C) Elution volumes of immune complexes saturated with a single Fab specificity (B) or combined Fab specificities (C) for determination of a standard curve for the calculation of Fab occupancy in immune complexes. Multiple dots represent different Fab-BG505 SOSIP.664 combinations with the same stoichiometry of binding. Note that complexes containing PGT151 and 35O22 were excluded from the right panel because of their aberrant elution behavior, which in the case of 35O22 only could be explained by a prolonged retention time of the Fabs themselves. See also Table S1.

**Figure 4:**
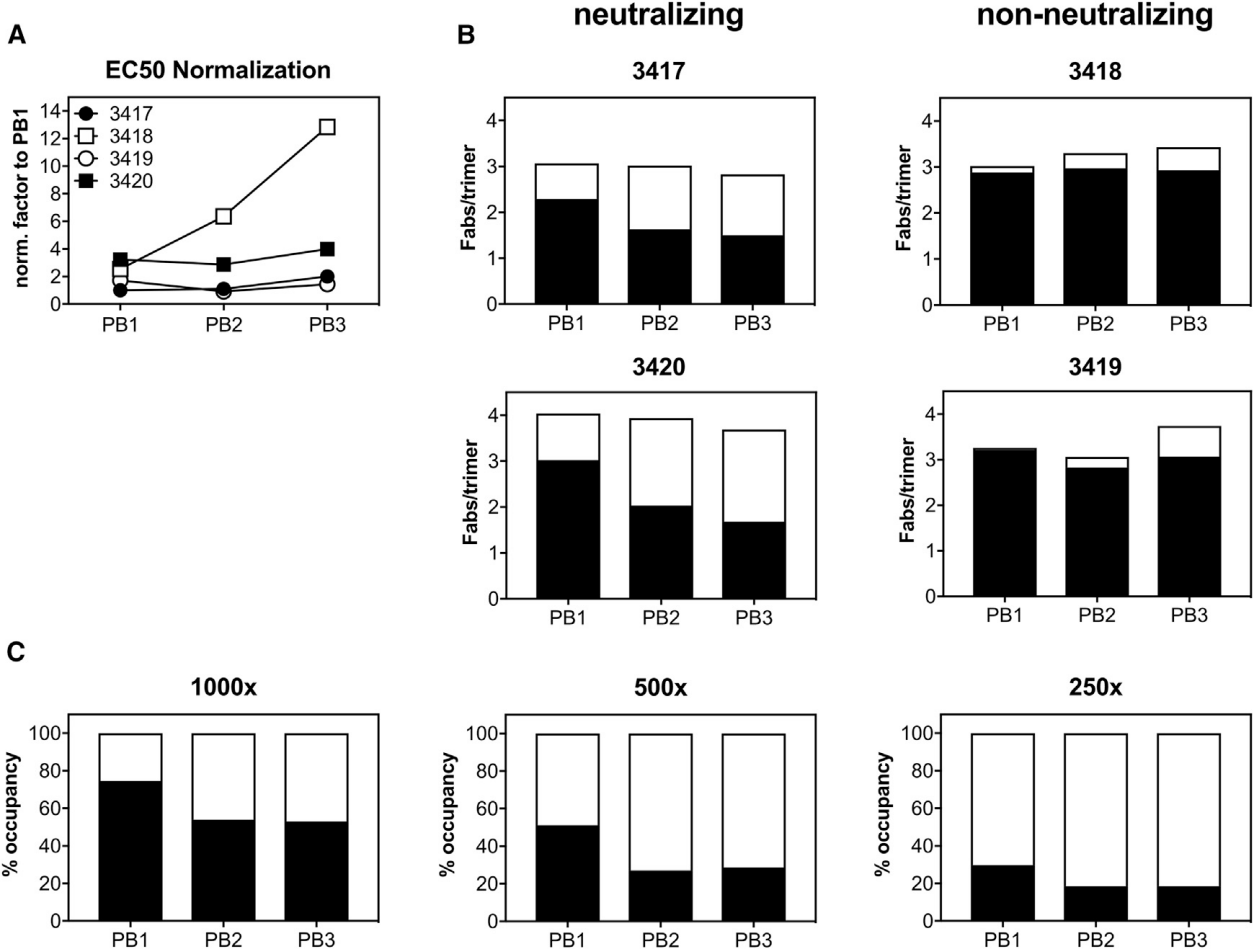
Analysis of 241 and 289 GH Binding Shows the Evolution of the Immune Response and Higher Affinities of GH-Specific Antibodies Than of Non-GH Antibodies Fab occupancy of immune complexes formed with rabbit polyclonal Fabs and BG505 SOSIP.664 with or without the GH was compared. (A) Factors used for normalization to rabbit 3417 PB1 EC_50_ were determined by ELISA. (B) Occupancy of GH-specific (white) and non-GH-specific (black) antibodies in immune complexes normalized to PB1-EC_50_ of rabbit 3417. (C) Comparison of relative affinities of GH-specific (white) and non-GH-specific (black) antibodies determined by measurement of Fab occupancy in complexes formed with titrated amounts of rabbit 3417 Fabs. See also Figure S2.

To independently confirm the findings made by nsEM, we used SEC to compare Fab occupancy in complexes formed with wild-type (WT) BG505 SOSIP.664 and a variant thereof, in which mutations S241N and P291S introduced N-linked glycosylation sites at positions N241 and N289, respectively. The 241 or 241 and 289 mutations have previously been shown to knock out the nAb response (Klasse et al., 2016, Klasse et al., 2018, McCoy et al., 2016), consistent with structural observations (McCoy et al., 2016). First, to determine the Fab occupancy in immune complexes, we digested a panel of well-characterized mAbs into Fabs and estimated their molecular weight by measuring retention volume by SEC. Most free Fabs eluted at around 18 mL from a Sepharose 6 Increase 10/300 column (Figure 3A). When immune complexes were formed between BG505 SOSIP.664 and saturating concentrations of a single Fab specificity, their previously determined stoichiometry of binding (Blattner et al., 2014, Julien et al., 2013a, Julien et al., 2013b, Lee et al., 2015, Liu et al., 2017, Stewart-Jones et al., 2016) was reflected in their elution volume (Figure 3B). However, complexes containing 35O22 or PGT151 Fabs were retained considerably longer than expected and were therefore excluded from further analysis. Finally, a large number of distinct immune complexes were formed and combined mAbs of different binding stoichiometries, and their elution volumes were determined (Table S1). These data enabled us to calculate a standard curve for estimating Fab occupancy directly from the elution volume of an immune complex (Figure 3C). To correct for the different amounts and affinities of BG505-specific antibodies present in the Fab preparation from the different bleeds, we determined EC_50_ values by ELISA (Figures 4A and S1A) and performed standardized immune-complex formation by overnight incubation of 10–25 μg of BG505 SOSIP.664, or variants thereof, with 2,000× the EC_50_ concentration of Fabs determined by ELISA. We subjected complexes to SEC to remove non-bound Fabs and estimate the average stoichiometry of Fabs bound to the immunogen. After SEC elution, complexes were subjected to single-particle nsEM.

To assess the occupancy, we formed immune complexes with Fab concentrations standardized to 2,000× the EC_50_ of 3417 PB1 by using BG505 SOSIP.664 and the GH-restored trimer (Figure 4). In rabbits that developed a neutralizing response, the fraction of N241- or N289-sensitive antibodies (i.e., GH specific) increased with each booster immunization (3417 and 3420; Figure 4B). By contrast, in rabbits that failed to mount nAbs (3418 and 3419), the antibody response was almost completely independent of the presence or absence of glycosylation at position 241 or 289, consistent with our nsEM findings.

To compare the overall affinities of the bound antibody classes, we used titrated Fabs from rabbit 3417 PB1 to form complexes with a fixed amount of WT BG505 SOSIP.664 or GH-filled trimer (Figure 4C) in non-saturating conditions. The fraction of GH-indifferent antibodies in the immune complexes decreased with each antibody dilution, indicating that these were of weaker affinity. Again, nsEM imaging of these complexes was consistent with these findings (2D classes for rabbit 3417 PB2 are shown as an example in Figure S2D).

To assess whether the lack of detection of V3 supersite or apex-specific antibodies was due to perturbation of these epitopes by binding of the predominant BOT or GH antibodies, we formed immune complexes by using a mixture of the BOT mAb 12N with either PG9 or PGT121 and found that binding of 12N did not affect binding of the other two mAbs in nsEM. Likewise, mixing 3417 PB1 Fabs containing BOT, COT, and GH antibodies with either PG9 or PGT121 did not prevent binding or detection of either mAb, as demonstrated by nsEM (Figure S1D). Finally, to determine whether the sera contained antibodies able to bind the V3 loop in the context of a well-ordered intact trimer, we first performed competition ELISA by using peptides corresponding to the V3 loop of BG505 SOSIP.664. Because no competition was detectable (Figure S1E), we concluded that only a minor fraction of Env trimer-specific antibodies was in fact against the V3 loop. We therefore enriched for V3-specific antibodies by affinity chromatography and obtained ∼1 μg of V3-specific Fabs per milligram of total Fabs. A fraction of these V3-specific Fabs were indeed able to bind immobilized BG505 SOSIP.664 in ELISA (Figure S1F) but were not observed in nsEM, most likely as a result of low affinity and fast off rates. In total, using nsEM and image analysis, we have discovered a class of antibodies that have not been described so far (COT), identified classes of antibodies that correlate with neutralization (GH1), and been able to expand our knowledge of the immunodominance of the BOT epitopes.

### 3D CryoEM Studies of Env-Fab Complexes Reveal High-Resolution Information about Targeted Epitopes

**Figure 5:**
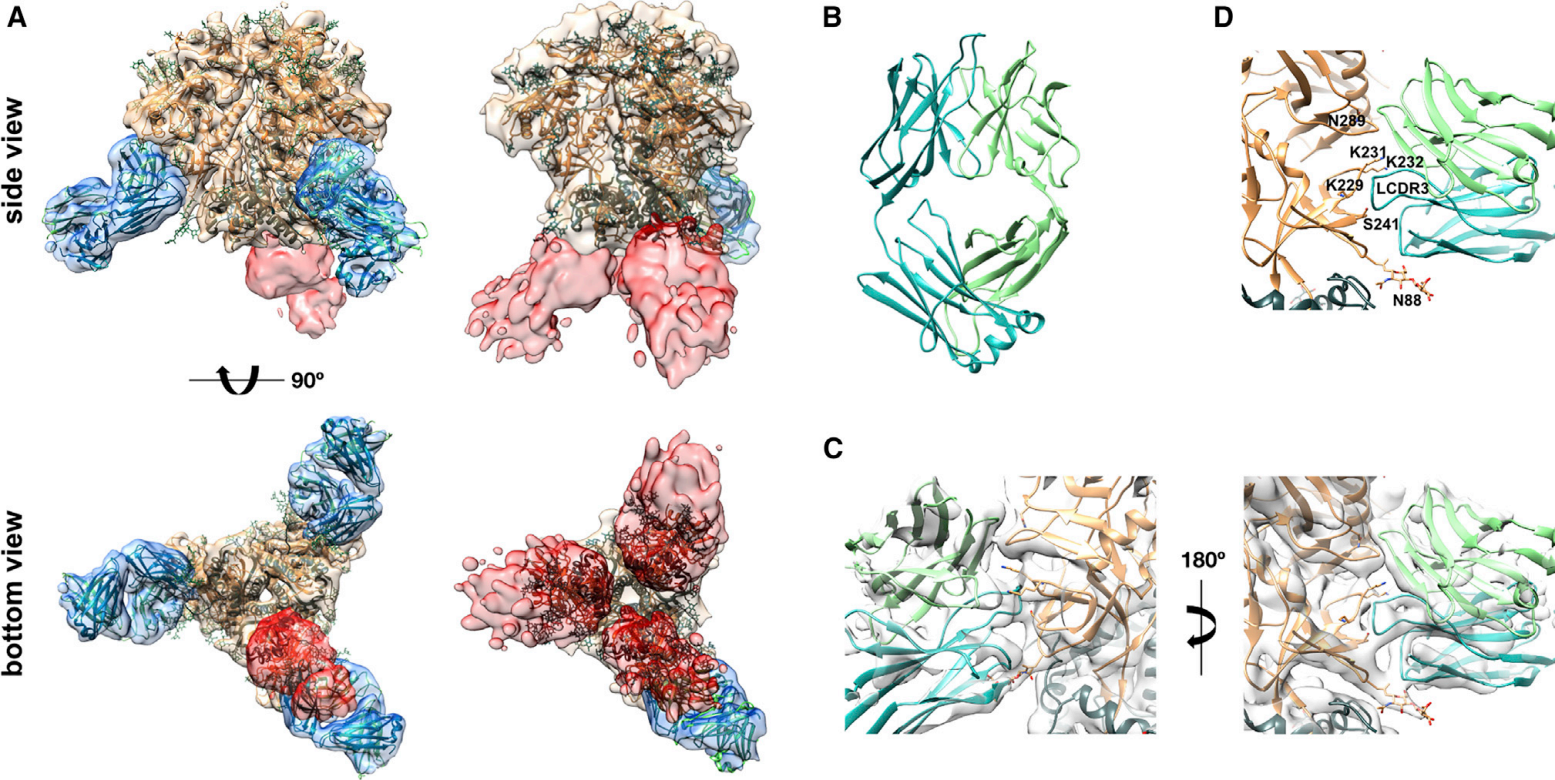
Analysis of a Polyclonal Immune Complex Structure Obtained by cryoEM with Sub-nanometer Resolution (A) Side and bottom views of two representative 3D reconstructions for immune complexes with Fabs originating from PB1 of rabbit 3417. EM densities are depicted in beige (Env), blue (GH binding Fabs), and red (BOT binding Fabs). The crystal-structure coordinates of BG505 SOSIP (PDB: 5I8H) were fitted into the EM densities and are depicted as a backbone in beige for gp120 and in gray for gp41. The 10A light chains are colored cyan, and heavy chains are colored green (cf. B–D). (B) Crystal structure of rabbit mAb 10A (PDB: 6CJK). The long LCDR3 extends away from the surface of the paratope. (C) Close-up views of a high-resolution cryoEM map of BG505 SOSIP.664 in complex with polyclonal Fabs. The cryoEM BG505 SOSIP.664 structure (PDB: 5ACO) and crystal structure of Fab 10A were fitted onto the map. (D) Close-up view of the epitope-paratope. The long LCDR3 makes the majority of contacts with Env in a lysine-rich loop directly above the S241 GH residue. Position N289, whose glycosylation would interfere with antibody binding (but is not glycosylated in BG505 SOSIP.664), is indicated in the structure. The glycan present at N88 is represented by sticks and exhibits density in the cryoEM map that interacts with the light chain. See also Figure S3 and Table S2.

Although nsEM and 2D classification were sufficient to identify the predominant epitope specificities elicited by BG505 SOSIP.664 immunization, we also attempted cryo-electron microscopy (cryoEM) and 3D reconstruction of complexes from one of the serum samples (rabbit 3417, PB1) for a more detailed analysis. We collected a dataset of ∼162,000 complexes and analyzed them by 2D classification. The results were consistent with the nsEM data, although as expected, the cryoEM data had a much great diversity of views of the complexes as a result of the free tumbling of particles in solution before rapid freezing, as well as a higher number of imaged particles. This relatively isotropic distribution of complexes is essential for robust 3D classification and reconstruction. We first calculated a single 3D reconstruction of the entire dataset by using CryoSparc (Punjani et al., 2017) (Figure S3), which included a heterogeneous mixture of Env-Fab complexes, resulting in a ∼4.7 Å resolution density map. The BG505 SOSIP.664 portion of this “global average” map was very well resolved, and further 3D classification and refinement resulted in four reconstructions that were resolved to sub-nanometer resolution. Previously solved atomic structures of the trimer could be fitted well into these maps, two of which are displayed in Figure 5A.

The main conclusion from the high-resolution analyses in CryoSparc is the high-quality density in the maps that corresponded to the GH1 antibodies, suggesting a structurally homogeneous mAb population. Further, when the high-resolution structure of Fab 10A, which we solved by X-ray crystallography (Figure 5B and Table S2; PDB 6CJK), was fit into the density map, it was very similar, particularly with regard to the main point of contact, the long LCDR3 loop (Figure 5C). Notably, in HCDR1, HCDR2, and HCDR3, the loop lengths of the polyclonal average appeared to differ from that of 10A, consistent with some variation in loop length and heavy-chain usage between these and 10A. These data suggest that the rabbits might have biased light-chain usage that preferentially targeted the GH epitope, whereby LCDR3 made the majority of contacts with Env in a lysine-rich loop directly above the S241 residue (Figure 5D). Moreover, connecting densities between the glycan at position N88 and the light chain suggest additional direct interactions (Figure 5C). Although the epitope-paratope regions were well resolved, for the C-terminal end of constant region 1, the 10A fit became poorer and densities become more diffuse, probably as a result of a slight difference in the positions of their C termini. Whether these differences reflected actual differences in the angle of approach of individual antibody species present in the polyclonal sera or whether this was due to the inherent flexibility of the hinge region of the Fabs could not be determined from the available data. In our previous study, we showed at low resolution that nAbs isolated from different rabbits targeted the GH in very similar manners (McCoy et al., 2016). Here, in our high-resolution cryoEM analysis, the close resemblance between the averaged polyclonal densities and the structure of mAb 10A, isolated from a different rabbit, again suggests highly convergent responses, even at the molecular level. Although we can only speculate at this point whether this observation was due to the dominance of a single clonotype or whether it was the result of convergent evolution of multiple clonotypes, the decreasing resolution of the GH1 density map in regions distal to the paratope indicates that the individual Fabs bound at varying angles of approach.

**Figure 6:**
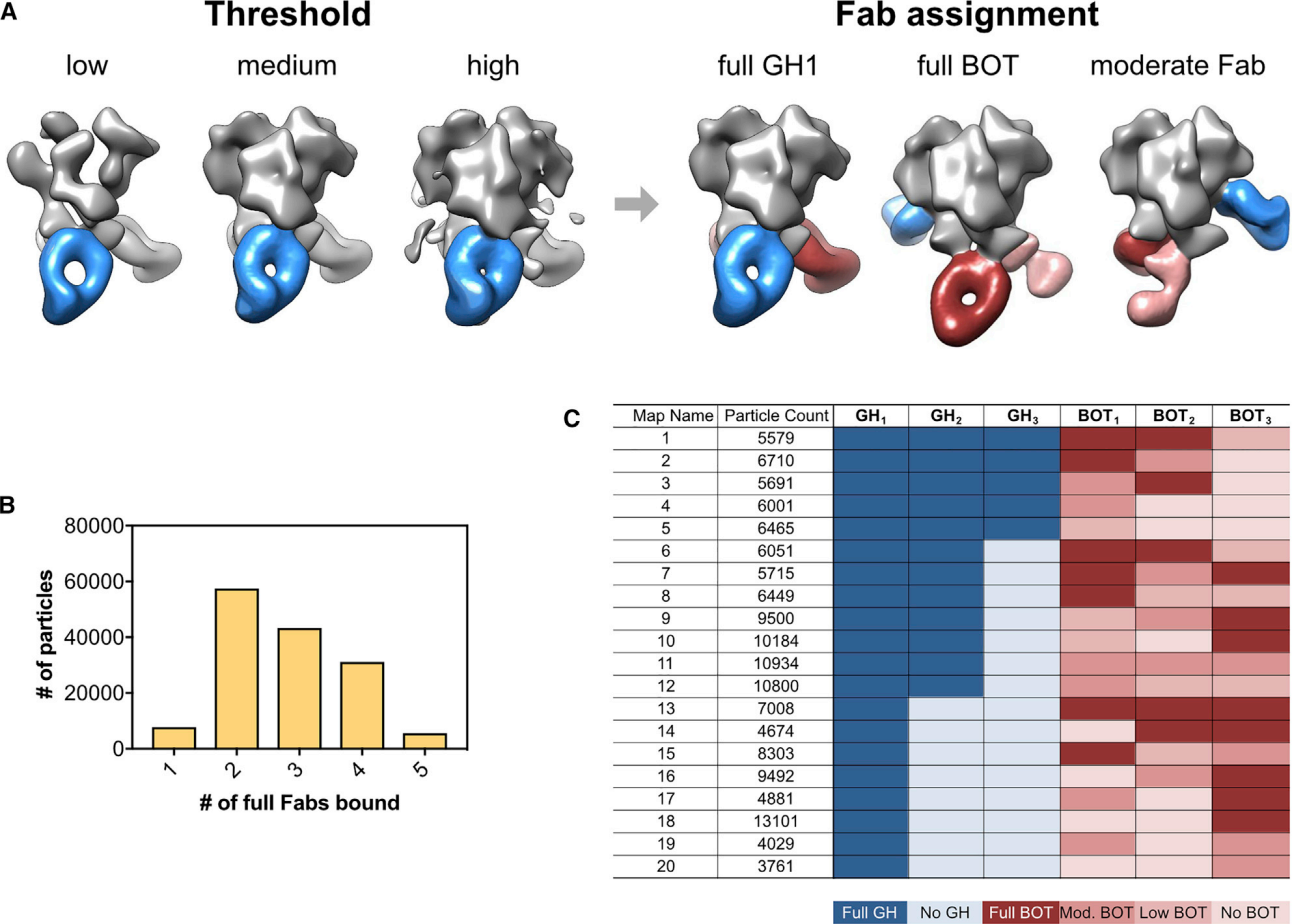
Semiquantitative Analysis of Epitope Occupancy in cryoEM 3D Reconstructions of Immune Complexes between BG505 SOSIP.664 and Fabs Originating from Rabbit 3417 PB1 (A) Examples of different occupancies for the indicated GH epitope. For normalizing thresholds of the individual 3D reconstructions, all density maps were overlaid with a reference structure for BG505 SOSIP.664 (EMD: 8312) and adjusted in volume threshold until the Env volumes were identical to those of the reference structure. Occupancy was then estimated, starting from the GH1 antibody present in all structures (referred to as GH_1_), in a clockwise direction. (B) Number of particles displaying the indicated full occupancy. (C) Number of particles containing full (darker colors), medium, and partial (lighter colors) occupancies for each of the indicated antibody-binding sites. See also Figure S3.

In the CryoSparc refinement, the complexes were not completely classified, and in all reconstructions, there was evidence of partial occupancy of one or more Fabs. The densities corresponding to Fabs varied between epitopes and provided a rough approximation of stoichiometry: densities that comprised the intact molecular volume of Fabs were consistent with high stoichiometry, whereas incomplete Fab volumes were consistent with lower stoichiometry but might also have reflected to some degree a higher flexibility or diversity of the bound Fabs (cf. BOT densities in Figure 5A). We therefore devised a strategy to estimate the occupancy on the basis of the Fab density apparent at different map thresholds. Using RELION (Scheres, 2012) on the same dataset, we undertook an exhaustive 3D classification approach to determine as many 3D reconstructions as possible given the size of the dataset (Figure S3). On the basis of this approach, we computationally isolated 20 complexes at modest resolution, which enabled us to observe the diversity of epitopes targeted and estimate the response to each epitope. Our 3D classification scheme allowed us to semiquantitatively report the total occupancy of the two primary antibody responses, BOT and GH (Figure 6). At the Fab concentration used, all immune complexes contained at least one GH1 Fab. The majority of 3D models displayed partial occupancy at one or two of the potential GH and BOT binding sites, and no other pattern was discernable. Quantification of 3D classes provided semiquantitative information for recognition of the different antibody classes. Moreover, by adding cryoEM analyses to nsEM, we gained structural information about the GH1 epitope recognition and demonstrated a structurally highly convergent evolution of these GH1-specific antibodies in rabbits.

## Discussion

Our polyclonal imaging approach using nsEM can provide snapshots of the antibody response at any given time after vaccination and has enabled us to extensively and semiquantitatively map the polyclonal immune response to a protein or glycoprotein immunogen and thereby shed light on the epitopic diversity and maturation of antibody responses in vaccinated animals. Our approach could equally be applied to follow the course of a response during natural infection. Using this approach, we confirmed GH1 class antibodies as major source of neutralization in BG505-SOSIP.664-immunized rabbits. In addition, we were able to discern differences in the kinetics by which the nAb responses are mounted and demonstrated that the lack of neutralization in this particular case was due to a failure to mount GH-specific antibodies. Moreover, we identified two additional classes of antibodies, namely GH2 and COT, that have not been previously described. The fact that GH2 antibodies can be found in samples with no neutralizing activity suggests that this class of antibody is non-neutralizing. COT antibodies could only be found in the neutralizing rabbits 3417 and 3420, but the absence of neutralization of a BG505 N332 virus with restored glycans at positions N241 (McCoy et al., 2016) and N289 indicates that this class of antibodies is either non-neutralizing or too rare to provide neutralization at least in these specific rabbits. Obtaining a definitive answer about their biological activity, however, will require additional experiments with mAbs from these classes. Besides identifying the predominantly elicited antibody classes and the most likely source for autologous neutralization, our rapid nsEM approach also provided approximate information about the affinity of the antibody classes detected. For example, we showed that early BOT class antibodies were inferior to GH-binding antibodies in that they disappeared from immune complexes at higher Fab concentrations than GH class antibodies, and this property did not change throughout the course of the immunization. Overall, our findings were consistent with and extend those of previously published rabbit-immunization studies (de Taeye et al., 2015, Klasse et al., 2016, McCoy et al., 2016, Sanders et al., 2015, Torrents de la Peña et al., 2017, Torrents de la Peña et al., 2018). Moreover, we also corroborated our nsEM data with occupancy data determined by a different, biochemical method. Successful epitope mapping experiments using sera from mice immunized with influenza A virus hemagglutinin and ongoing studies investigating the humoral response to BG505 SOSIPs in non-human primates (NHPs) (both will be published elsewhere) further indicate that this method is not restricted to rabbit immunoglobulin but can be applied to other species and other antigens as well.

The addition of cryoEM to our methodology enabled us to obtain sub-nanometer-resolution 3D reconstructions that provided us with additional insights into the molecular recognition of the GH1 epitope on the immunogen. We detected a high degree of structural conservation in these antibodies, in particular for LCDR3. Our data, however, do not allow us to differentiate whether this was due to the dominance of a single antibody clonotype or whether different antibody clones converged to structurally similar paratopes.

Although the images might not recapitulate the full diversity of antigen-specific antibodies in the serum, they most likely represent a snapshot of the predominantly recognized epitopes. Thus, our workflow and a small set of SOSIP trimer constructs allow the rapid derivation of information for assessing ongoing immunization experiments and provide data for immunogen redesign. For example, the early dominant response to the trimer base neo-epitope is concerning because the activation and proliferation of BOT-specific B cells might restrict resources for B cells recognizing more productive and neutralizing epitopes. Furthermore, nearly all of the antibodies bind at an upward angle relative to the trimer. This contrasts with all known bnAbs that bind at a downward or parallel angle of approach, which reflects the fact that the soluble BG505 SOSIP.664 can also be presented to rabbit B cells in an “upside-down fashion” relative to virion presentation. Therefore, particulate display of BG505 SOSIP.664 trimers could prevent presentation of the base and lower epitopes in Env and improve immune responses such that they more closely resemble nAb and, perhaps even to some extent, bnAb responses. Overall, the rabbit responses to BG505 SOSIP.664 trimers appear quite narrow and are limited to a few epitopes. In our images, we did not observe V3 loop, non-neutralizing Abs, which have been reported in ELISA binding assays (McCoy et al., 2016, Pauthner et al., 2017, Sanders et al., 2015). Because we formed complexes in solution by using fully native Env trimer proteins that do not expose the V3 loop or other epitopes that can become exposed after immobilization, this is perhaps not surprising. Our approach therefore preferentially detects the most relevant responses to the surface of pre-fusion conformation of Env. Although there is some indication from the COT class of antibodies that it might be more challenging for our method to detect less frequent antibody classes, they still did not go undetected. Additional complexes formed with epitope-knockout variants of the immunogen or the use of pre-adsorbed Fab preparations could also aid in the detection of rarer specificities.

In line with previous findings, our study also showed that the appearance and maintenance of epitope specificities can vary. The EM imaging provides predictive models that accurately anticipate neutralization on the basis of the epitopes targeted, at least in rabbits. Preliminary analysis using conventional epitope mapping of NHPs immunized with BG505 SOSIP.664 (Pauthner et al., 2017) has found a more diverse response than in rabbits. Our polyclonal epitope-mapping approach could be used for a more comprehensive analysis of such NHP samples as well as human responses to vaccines, which in turn would inform prime-boosting vaccination strategies. For example, direct visualization by nsEM could be used for rapidly deciding whether a single prime is sufficient before the introduction of a heterologous boost or whether a second prime should be given if a desired antibody specificity cannot be observed after the first immunization. Further, one can determine whether the elicited polyclonal antibodies directly (via epitope overlap) or indirectly (through steric blockade) interfere with an intended epitope-focused response. Finally, comparison of imaging from human and animal model studies will reveal the similarities and differences in responses between humans and animal models and help determine the relative value of different preclinical studies and the most appropriate animal model for iterative vaccine design, including different immunization regimens and adjuvants.

## STAR★Methods

### Key Resources Table

**Table:** 

REAGENT or RESOURCE	SOURCE	IDENTIFIER
**Antibodies**		

HRP-conjugated anti-rabbit IgG, F(ab’)_2_ specific	Jackson ImmunoResearch	Cat# 111-035-047
HRP-conjugated anti-human IgG, F(ab’)_2_ specific	Jackson ImmunoResearch	Cat# 109-035-097
Monoclonal anti-HIV-1 Env PGT121	Produced in house (Walker et al., 2011)	RRID: AB_2491041
Monoclonal anti-HIV-1 Env PGT145	Produced in house (Walker et al., 2011)	RRID: AB_2491054
Monoclonal anti-HIV-1 Env PGT151	Produced in house (Falkowska et al., 2014)	N/A
Monoclonal anti-HIV-1 Env PG9	Produced in house (Walker et al., 2009)	RRID: AB_2491030
Monoclonal anti-HIV-1 Env PG16	Produced in house (Walker et al., 2009)	RRID: AB_2491031
Monoclonal anti-HIV-1 Env PGDM1400	Produced in house (Sok et al., 2014)	N/A
Monoclonal anti-HIV-1 Env PGV04	Produced in house (Wu et al., 2011)	N/A
Monoclonal anti-HIV-1 Env VRC01	Produced in house (Wu et al., 2010)	RRID: AB_2491019
Monoclonal anti-HIV-1 Env 10-1074	Produced in house (Shingai et al., 2013)	RRID: AB_2491062
Monoclonal anti-HIV-1 Env 35O22	Produced in house (Huang et al., 2014)	N/A
Monoclonal anti-HIV-1 Env 3BC315	Produced in house (Klein et al., 2012)	N/A
Monoclonal anti-HIV-1 Env 10A	Produced in house (McCoy et al., 2016)	N/A
Monoclonal anti-HIV-1 Env 11A	Produced in house (McCoy et al., 2016)	N/A
Monoclonal anti-HIV-1 Env 12A	Produced in house (McCoy et al., 2016)	N/A
Monoclonal anti-HIV-1 Env 12N	Produced in house (McCoy et al., 2016)	N/A
Monoclonal anti-HIV-1 Env 14e	Produced in house (Sanders et al., 2013; Prof. James Robinson)	N/A
Monoclonal anti-HIV-1 Env 19b	Produced in house (Robinson et al., 1990)	N/A

**Bacterial and Virus Strains**		

BG505 N332 HIV-1 Env-pseudotyped virus	Produced in house (Pauthner et al., 2017)	N/A
BG505 N241/289/332 HIV-1 Env-pseudotyped virus	Produced in house (Pauthner et al., 2017)	N/A

**Biological Samples**		

Plasma from rabbits 3417–3420	McCoy et al., 2016	N/A

**Chemicals, Peptides, and Recombinant Proteins**		

BG505 SOSIP.664	Produced in house (Sanders et al., 2013)	N/A
BG505 SOSIP.664 N241 N289	Produced in house (Sanders et al., 2013)	N/A
BG505 SOSIP.664 v4.1	Produced in house (de Taeye et al., 2015)	N/A
BG505 SOSIP.664 v5.2	Produced in house (Torrents de la Peña et al., 2017)	N/A
BG505 MD39 CPG9	Prof. William R. Schief (Kulp et al., 2017)	N/A
Immobilized Papain	Thermo Fisher Scientific	Cat# 20341
Immobilized IdeS (Fabricator)	Genovis	Cat# A0-FR6-100
Immobilized protein A	GE Healthcare	Cat# 17-5280-02
Immobilized protein G	GE Healthcare	Cat# 17-0618-05
Immobilized streptavidin (high capacity)	Thermo Fisher Scientific	Cat# 20357
NeutrAvidin	Thermo Fisher Scientific	Cat# 31000
InstantBlue Coomassie stain	Expedeon	Cat# ISB1L
Desalting columns	Thermo Fisher Scientific	Cat# 89890
TMB substrate	Thermo Fisher Scientific	Cat# 34028
Polyethylenimine (PEI) HCl MAX, Linear, Mw 40,000	Polysciences	Cat# 24765-1
TMB substrate	Thermo Fisher Scientific	Cat# 34028
DEAE-Dextran	Sigma-Aldrich	Cat# D9885-10G
X-tremeGENE 9 DNA Transfection Reagent	Sigma-Aldrich	Cat# XTG9-RO
Biotinylated BG505 V3 peptide	GenScript	N/A
Uranyl Formate	Electron Microscopy Sciences	Cat# 22451
n-Dodecyl-β-D-Maltopyranoside (DDM)	Anatrace	Cat# D310 25 GM

**Critical Commercial Assays**		

Pierce Fab Preparation Kit	Thermo Fisher Scientific	Cat# 44985
BirA biotin-protein ligase standard reaction kit	Avidity	Cat# BirA500
Bright-Glo Luciferase Assay System	Promega	Cat# E2610
Bolt 4-12% Bis-Tris Plus Gels	Thermo Fisher Scientific	Cat# NW04127BOX
Negative stain EM Grids	Electron Microscopy Sciences	Cat# EMS400-CU
CryoEM grids	Electron Microscopy Sciences	Cat# Q26194

**Deposited Data**		

CryoEM map of polyclonal serum in complex with BG505 SOSIP.664 from rabbit 3417 at post boost 2. All particles from 2D classification went into this Ab-initio reconstruction made in CryoSparc.	EMDataBank	EMD: 7552
CryoEM map of polyclonal serum in complex with BG505 SOSIP.664 from rabbit 3417 at post boost 2. Refined map after a second round of classification and removing a class shown to have only one glycan hole (GH) Fab bound.	EMDataBank	EMD: 7553
CryoEM map of polyclonal serum in complex with BG505 SOSIP.664 from rabbit 3417 at post boost 2. Refined class after two subsequent classifications showed no significant differences in 3D maps.	EMDataBank	EMD: 7554
CryoEM map of polyclonal serum in complex with BG505 SOSIP.664 from rabbit 3417 at post boost 2. Refined map after adding particles from a separate class shown to have a single GH Fab bound.	EMDataBank	EMD: 7555
CryoEM map of polyclonal serum in complex with BG505 SOSIP.664 from rabbit 3417 at post boost 2. Same thing as EMD-7446.	EMDataBank	EMD: 7557
Negative stain EM map of BG505 SOSIP.664 in complex with PG9 and 12N Fab.	EMDataBank	EMD: 7570
Negative stain EM map of BG505 SOSIP.664 in complex with PGT121 and 12N Fabs.	EMDataBank	EMD: 7903
Negative stain EM map of BG505 SOSIP.664 in complex with PGT121 and polyclonal serum from rabbit 3417 at post post boost 1.	EMDataBank	EMD: 7904
Negative stain EM map of BG505 SOSIP.664 in complex with PG9 and polyclonal serum from rabbit 3417 at post post boost 1.	EMDataBank	EMD: 7906
Negative stain EM map of polyclonal serum in complex with BG505 SOSIP.664 from rabbit 3418 PB2. Rabbit was not protected.	EMDataBank	EMD: 7887
Negative stain EM map of polyclonal serum in complex with BG505 SOSIP.664 from rabbit 3418 PB2. Rabbit was not protected.	EMDataBank	EMD: 7888
Negative stain EM map of polyclonal serum in complex with BG505 SOSIP.664 from rabbit 3419 PB2. Rabbit was not protected.	EMDataBank	EMD: 7889
Negative stain EM map of polyclonal serum in complex with BG505 SOSIP.664 from rabbit 3419 PB2. Rabbit was not protected.	EMDataBank	EMD: 7890
Negative stain EM map of polyclonal serum in complex with BG505 SOSIP.664 from rabbit 3417 PB2.	EMDataBank	EMD: 7891
Negative stain EM map of polyclonal serum in complex with BG505 SOSIP.664 from rabbit 3417 PB2.	EMDataBank	EMD: 7892
Negative stain EM map of polyclonal serum in complex with BG505 SOSIP.664 from rabbit 3420 PB2.	EMDataBank	EMD: 7893
Negative stain EM map of polyclonal serum in complex with BG505 SOSIP.664 from rabbit 3420 PB2.	EMDataBank	EMD: 7894
Negative stain EM map of polyclonal serum in complex with BG505 MD39 CPG9.	EMDataBank	EMD: 7895
CryoEM map of polyclonal serum in complex with BG505 SOSIP.664 from rabbit 3417 at post boost 2. All particles from 2D classification went into this Ab-initio reconstruction made in CryoSparc and C3 symmetry applied. This map was used to dock atomic models of BG505 SOSIP.664 trimer (orange, PDB: 5V8M) and Fab 10A (blue, PDB: 6CJK) to create a hybrid model.	EMDataBank	EMD: 7896; PDB: 6DID
Fab 10A X-ray crystal structure	RSCB Protein Data Bank	PDB: 6CJK

**Experimental Models: Cell Lines**		

Human: TZM-bl	NIH AIDS Reagent Program	Cat# 8129
Human: FreeStyle HEK293F	Thermo Fisher Scientific	Cat# R79007
Human: HEK293T	ATCC	Cat# CRL-3216

**Experimental Models: Organisms/Strains**		

Rabbit: New Zealand white	Western Oregon Rabbit	N/A

**Software and Algorithms**		

Prism v7.0	GraphPad	https://www.graphpad.com
Unicorn 7.0	GE Healthcare	http://www.gelifesciences.com/
UCSF Chimera	Pettersen et al., 2004	N/A
Appion database	Lander et al., 2009	N/A
Leginon	Suloway et al., 2005	N/A
DoG Picker	Voss et al., 2009	N/A
Relion	Scheres, 2012	N/A
CryoSparc	Punjani et al., 2017	N/A

### Contact for Reagent and Resource Sharing

Further information and requests for resources and reagents should be directed to and will be fulfilled by the Lead Contact, Lars Hangartner (lhangart@scripps.edu).

### Experimental Model and Subject Details

#### Rabbits

The samples used in this study derived from the previously described immunization of animals 3417, 3418, 3419, and 3420 (McCoy et al., 2016). Briefly, 15-week-old female New Zealand white rabbits were immunized twice with liposomes embedded with BG505 SOSIP.664 v3.2, and then three soluble BG505 SOSIP.664 v3.2 protein boosts. The Scripps Research Institute (TSRI) Institutional Animal Care and Use Office and the Committee (IACUC) approved all experimental procedures involving rabbits 3409–3420. All procedures were performed by TSRI Department of Animal Resources (DAR) staff in accordance with IACUC protocol 14-0002.

### Cell Lines

TZM-bl cells (human female HeLa-derived cancer cell line) were maintained at 37°C and 5% CO_2_ in high glucose Dulbecco’s Modified Eagle Medium (DMEM, Corning) containing 1X Penicillin-Streptomycin (Corning), 2 mM L-Glutamine (Corning), and 10% heat-inactivated fetal bovine serum (FBS, Omega Scientific).

### Method Details

#### Anti-HIV-1 *Env* Monoclonal Antibodies

Monoclonal antibodies (mAbs) were expressed by co-transfection of HEK293F cells (Thermo Fisher Scientific). Briefly, 156 μg heavy chain and 156 μg light chain-expressing plasmids were mixed in 25 mL Opti-Minimum Essential Media (MEM), and then added to 25 mL Opti-MEM containing 937.5 μg Polyethylenimine (PEI) MAX 40,000 (Polysciences). After 30 min at room temperature (RT), the DNA/PEI mix was added to 10^9^ HEK293F cells in 1 L FreeStyle 293 Expression Medium (Thermo Fisher Scientific), and further incubated for 6–7 days at 37°C, 8% CO_2_, 80% humidity, 135 rpm. Cells were then pelleted by centrifugation and filtered through 0.22 μm Rapid-Flow filter units (Nalgene). Filtered supernatant was applied to a column containing a 1 mL packed Protein G Sepharose Fast Flow (GE Healthcare) equilibrated with phosphate-buffered saline (PBS). The column was washed with 20 column-volumes of PBS, and mAb eluted with 0.1 M glycine pH 2.5 in a 1:10 volume of 1 M Tris-HCL pH 8 solution. Antibodies were concentrated and buffer-exchanged into PBS using 10,000 MWCO Amicon Ultra-15 centrifugal filter units (EMD Millipore) over three rounds of spinning.

His-tagged Fab 10A was recombinantly expressed and secreted as a soluble protein in HEK293F cells. The supernatant was concentrated and loaded onto a Ni-NTA affinity column, and the Fabs were eluted using an imidazole gradient. Next, Fabs were loaded onto a cation exchange column (monoS) and eluted using a salt gradient. Fractions containing pure Fab were pooled, concentrated, and buffer exchanged into tris-buffered saline (TBS) buffer (50 mM Tris, 150 mM NaCl, pH 7.5).

#### Soluble *Env* Protein Production

BG505 SOSIP.664 v3.2 (Sanders et al., 2015), BG505 SOSIP.664 v4.1 (de Taeye et al., 2015), BG505 SOSIP.664 v5.2 (Torrents de la Peña et al., 2017), BG505 SOSIP.664 v5.2 with glycans at positions N241 and N289 (will be published elsewhere), or BG505 MD39 CPG9 (Kulp et al., 2017) (with or without C-terminal Avi or Strep tag to enable biotinylation and purification) were used in this study. Compared to BG505 SOSIP.664 v3.2, BG505 SOSIP.664 v4.1 contains a A316W mutation, which improves hydrophobic packing and stability of the V3 loop, and an E64K mutation, which reduces spontaneous sampling of the CD4-bound “open” trimer conformation. The BG505 SOSIP.664 v5.2 is similar to the v4.1 design, with the addition of a second disulfide bond between gp120 (A73C) and gp41 (A561C) to further increase trimer stability. BG505 MD39 CPG9 contains the MD39 stabilizing mutations (Steichen et al., 2016), glycans at positions N80, N241, N289, N630, and a glycosylated loop connecting gp120 and gp41 that block binding to the bottom of the trimer. BG505 trimers were expressed in HEK293F cells by transient co-transfection with furin (except for BG505 MD39 CPG9, which is cleavage independent), and then purified using methods described elsewhere (Pugach et al., 2015), with either 2G12 or PGT145-affinity columns followed by size exclusion chromatography (SEC). Fractions corresponding to trimer were pooled and concentrated down to 1–2 mg/mL. Avi-tagged proteins were biotinylated after 2G12 or PGT145-affinity columns using the BirA biotin-protein ligase standard reaction kit (Avidity) under the following conditions and reagents from the kit: 100 μL of Avi-tagged protein, 15 μL of 10× Biomix B, 15 μL of BIO200, 15 μL of 1 M Tris-HCL pH 8, 5 μL of BirA enzyme, incubated for 1 hr at 37°C. Excess biotin and BirA enzyme was finally removed by SEC. All samples were sterile filtered prior to aliquoting and flash freezing. Structural validation of trimers was performed by analysis of negative-stain electron microscopy (EM) 2D class averages. The proteins used for immunizations had no His-tag.

#### Plasma or Serum IgG Purification

IgGs were purified from plasma or serum of immunized animals using protein A and/or G Sepharose resin (GE Healthcare), at a ratio of 1 mL packed resin for each mililiter of undiluted plasma or serum. Samples were diluted at least 4-fold in PBS, then incubated with protein A/G resin for 5 hr at RT or overnight at 4°C. The resin was washed 3 times with 10 volumes PBS, and the IgGs eluted with 5–10 volumes of 0.1 M glycine pH 2.5 immediately neutralized with 1 M Tris-HCL pH 8. Buffer was exchanged to PBS or TBS either by dialysis or by centrifugation using 10 kDa cutoff membranes (Thermo Fisher Scientific) or tubes (EMD Millipore), respectively.

#### Fab Preparation

Fabs were prepared for EM imaging. To make Fab, IgG were digested with papain-agarose resin (Thermo Fisher Scientific) for 5 hr at 37°C using 50 μl settled resin/mg IgG in 20 mM sodium phosphate, 10 mM EDTA, 20 mM cysteine, pH 7.4. Fc and non-digested IgG were removed by 1 hr incubation at RT with protein A Sepharose resin (GE Healthcare), using 0.2 mL packed resin/mg of initial IgG. After protein A incubation, cysteine was removed from the flow-through containing the digested Fab by dialysis or by ultracentrifugation using 10 kDa cutoff (Thermo Fisher Scientific) or tubes (EMD Millipore), respectively.

#### Fab Quality Control by SDS-PAGE and SEC

Fab size and homogeneity were assessed by Sodium Dodecyl Sulfate - PolyAcrylamide Gel Electrophoresis (SDS-PAGE) and SEC. For SDS-PAGE, 5 μg protein/lane was loaded on a 4%–12% Bolt Bis-Tris Plus gel (Thermo Fisher Scientific) in reducing VS non-reducing conditions, and run at 200 V in 3-Morpholinopropane-1-sulfonic acid (MOPS) buffer. Bands were visualized with Coomassie staining (Expedeon), and the size of the fragments evaluated by running a protein standard ladder (Thermo Fisher Scientific). For SEC, 50 μg protein was loaded on a Superdex 200 increase 10/300 column using a 100 μL loop, and run at 0.5 mL/min using an Äkta Pure system (GE Healthcare). Fab peaks were analyzed with the provided Unicorn 7.0.2 software. The size of the fragments was estimated with the help of a linear regression calculated by running a mix of proteins with known molecular weight (BioRad) on the same column.

#### BG505 ELISA

High-binding enzyme-linked immunosorbent assay (ELISA) plates (Thermo Fisher Scientific) were coated with neutravidin (Thermo Fisher Scientific) or a BG505-binding antibody (mostly human PG9, PGT145, or PGT121, or rabbit 10A or 12N) overnight at 4°C, then blocked with 3% BSA for 2 hr at RT. Biotinylated or untagged BG505 SOSIP.664 was captured on the neutravidin/antibody plate for 2 hr at RT, before adding serial dilutions of Fab or F(ab’)_2_ for additional 2 hr at RT. Binding of BG505-specific antibodies was assessed by Fab-specific secondary-horseradish peroxidase (HRP) antibodies (Jackson ImmunoResearch) after 1 hr incubation at RT. HRP activity was measured by adding 3,3′,5,5′-Tetramethylbenzidine (TMB)-substrate (Thermo Fisher Scientific), and blocking the reaction with 2 N sulfuric acid after 3 min incubation. OD450 was finally measured using a BioTek Synergy 2 plate reader (Perkin Elmer), and the effective concentration (EC), EC_50_ and EC_90,_ calculated using Prism 7 software (GraphPad). Relative abundance of BG505-specific antibodies was estimated by comparing the EC_50_s with those obtained from a total IgG ELISA. Competition with BG505 V3-peptide (TRPNNNTRKSIRIGPGQAFYATGDIIGDIRQAH, GenScript) was performed by pre-incubation of antibodies or plasmas with 150 μg/mL V3-peptide at room temperature for 1 hr before incubation on BG505 SOSIP.664 coated ELISA plates.

#### V3-Peptide ELISA

High-binding enzyme-linked immunosorbent assay (ELISA) plates (Thermo Fisher Scientific) were coated with neutravidin (Thermo Fisher Scientific) overnight at 4°C, then blocked with 3% BSA for 2 hr at RT. Biotinylated BG505 V3-peptide (TRPNNNTRKSIRIGPGQAFYATGDIIGDIRQAH, GenScript) was captured on the neutravidin plate for 2 hr at RT, before adding serial dilutions of Fab or plasma for additional 2 hr at RT. Binding of BG505 V3-peptide specific antibodies was assessed by Fab-specific secondary-horseradish peroxidase (HRP) antibodies (Jackson ImmunoResearch) after 1 hr incubation at RT. HRP activity was measured by adding 3,3′,5,5′-Tetramethylbenzidine (TMB)-substrate (Thermo Fisher Scientific), and blocking the reaction with 2 N sulfuric acid after 3 min incubation. OD450 was finally measured using a BioTek Synergy 2 plate reader (Perkin Elmer), and the effective concentration (EC), EC_50_ and EC90, calculated using Prism 7 software (GraphPad). Competition with BG505 V3-peptide (TRPNNNTRKSIRIGPGQAFYATGDIIGDIRQAH, GenScript) was performed by pre-incubation of antibodies or plasmas with 150 μg/mL V3-peptide at room temperature for 1 hr before incubation on BG505 SOSIP.664 coated ELISA plates.

#### Neutralization Assays

Replication incompetent HIV pseudovirus was produced by co-transfecting *env* plasmids with an *env*-deficient backbone plasmid (pSG3Δ*env*) in HEK293T cells in a 1:2 ratio, using the X-tremeGENE 9 transfection reagent (Roche). Pseudovirus was harvested after 48–72 hr by sterile-filtration (0.22 μm) of cell culture supernatants and titrated on TZM-bl cells. Neutralization was then assessed by TZM-bl assay: previously titrated pseudovirus were incubated with Fab for 1 hr at 37°C, and then transferred in a white 384-well plate (Greiner Bio-One) together with an equal volume of TZM-bl cells (4,000/well) resuspended in complete DMEM + 20 μg/mL Diethylaminoethyl (DEAE)-dextran. After 48 hr at 37°C and 5% CO_2_, the supernatant was removed and the cells lysed with Glo lysis buffer (Promega) for 5 min at RT. Luciferase activity was measured by the addition of Bright-Glo luciferase-substrate (Promega), and the luminescence signal read using a BioTek Synergy 2 plate reader. Full IgG and F(ab’)_2_ were used as control, and uninfected cells to correct for background.

#### Occupancy Standard Curve

Twelve-molar excess (for each mAb) of a single or combination of mAbs known to bind with different stoichiometries were incubated with 10 μg BG505 trimers overnight at RT in 100 μL total volume. Complexes were then run on a Superose 6 increase 10/300 column and Äkta Pure system (GE Healthcare) and the different elution peaks used to calculate a stoichiometry standard curve using the Prism 7 software (GraphPad).

#### Complexes for EM

10 μg BG505 trimers were incubated overnight at RT with 2000-fold EC_50_ excess of Fab in 100 μL total volume, and the complexes were then purified on a Superose 6 increase 10/300 column and Äkta Pure system (GE Healthcare) in TBS buffer. The fractions containing the complexes were pooled in 10 kDa cutoff tubes (EMD Millipore) and concentrated down to 50 μL final volume.

#### X-Ray Crystallography

Fab 10A was crystallized from solutions containing 10 mg/mL Fab in TBS buffer. Crystals were grown using sitting drop vapor diffusion with a well solution containing 0.1 M sodium citrate pH 5.26, 0.17 M ammonium acetate, 15% glycerol and 19% PEG4000. Crystals were grown at 298 K and appeared within 3 days. Fab 10A crystals were cryoprotected by soaking in a well solution supplemented with 30% glycerol. Diffraction data were collected at the Advanced Photon Source (APS) beamline 23ID-D. Data collection and processing statistics are outlined in Table S2. Datasets were indexed, integrated, and scaled using the HKL-2000 package (Otwinowski and Minor, 1997). The structures were solved by molecular replacement using PHASER (McCoy et al., 2007) with homology models for Fab 10A (SWISS-MODEL; Arnold et al., 2006, Biasini et al., 2014, Bordoli et al., 2009) as search models and further refined using phenix.refine (Adams et al., 2010) combined with manual building cycles in Coot (Emsley et al., 2010). Structure deposited: PDB: 6CJK.

#### Negative-Stain EM

SEC purified complexes were deposited at approximately 0.04 mg/mL onto carbon-coated copper grids and stained with 2% (w/v) uranyl formate (Briganti and Mauro, 1979) for 30 s as previously described (Pugach et al., 2015). Grids were imaged at 120 KeV using a Tecnai Spirit using Leginon (Suloway et al., 2005). Images were collected on a 4kx4k TemCam F416 detector and transferred into the Appion database (Lander et al., 2009) for initial image processing. Particles were picked using DoG Picker (Voss et al., 2009) and 2D classes were generated using MSA/MRA (Ogura et al., 2003). Particles corresponding to Env-Fab complexes were selected and further processed via 3D classification in Relion (Scheres, 2012) to separate out the unique complexes within the heterogeneous dataset before final refinement of each map. Figures were prepared using UCSF Chimera (Pettersen et al., 2004).

#### Cryo EM

The 3417 PB1 sample was concentrated to 5.6 mg/mL. Immediately before deposition onto a 1.2/1.3 200 Quantifoil grid (EMS) that were glow discharged for 10 s, 3μL of the concentrated complex was mixed with 1 μL of 0.42 mM Dodecyl Maltoside (DDM, Anatrace). Addition of DDM promoted appearance of complexes into holes and improved angular sampling of individual complexes. Grids were then blotted and plunged into liquid ethane using a Vitrobot (FEI) to capture complexes in vitreous ice. Cryo grids were transferred into a 200 KeV Talos Artica and images recorded on a 3710 × 3838 pixel Gatan K2 Summit detector using Leginon (Suloway et al., 2005) at a defocus range of −1.5 μm to −2.5 μm. Images were transferred to the Appion (Lander et al., 2009) database and particles were picked using DoG Picker (Voss et al., 2009) and placed into a stack. Initial 2D classification was conducted in Relion and non-Env particles were removed, creating a clean stack of 161,639 particles that was then subjected to 3D classification. The first round of 3D classification resulted in six reconstructions. Subsequent rounds of sequential 3D classification were conducted as illustrated in Figure S3. 3D reconstructions with similar occupancy of bound Fabs were combined before final refinement. This approach resulted in 20 unique 3D reconstructions that were then used to quantify the Fabs at each epitope.

The same cryo-particle stack was also subjected to image processing in CryoSparc (Punjani et al., 2017) that resulted in 4 subnanometer resolution reconstructions (Figure S3). We also calculated a global average of all particles that was resolved to 4.71 Å resolution.

#### Fab Occupancy Analysis

Within the 20 Relion cryoEM maps, there was still obvious sub-stoichiometric occupancy of Fabs at different epitopes. We derived a method to approximate the occupancy of the Fabs at each site to get a better estimate of the total response per epitope. In the end, each epitope was characterized as having full, moderate, low, or no occupancy using the following approach. Each reconstruction was normalized to best match the trimer density of a 20 Å resolution low pass filtered map of BG505 SOSIP.664. In every map, at these normalized thresholds, we saw at least one nicely resolved GH Fab. At this contour level, there were Fabs present at other epitopes that only had partial density. We then increased the density threshold (higher sigma density) and observed that the GH Fab density persisted; thus, we considered this to be fully occupied. Conversely, the partial density Fabs would disappear at this higher threshold, and we therefore considered this to be partial occupancy. To detect even lower occupancy, we decreased the threshold (lower sigma signal) relative to the normalized map and if density appeared at an epitope that began to resemble a Fab we considered this to be low occupancy. If no density appeared, then we characterized this as no occupancy. Our results are summarized in Figure 6.

### Quantification and Statistical Analysis

Statistical models inherent to Relion (Scheres, 2012) and CryoSparc (Punjani et al., 2017) were employed in image analysis to derive 2D classes and 3D models. Estimation of Fab occupancy in the 3D models was undertaken manually based on density thresholds in the 3D EM reconstructions as described in the method details under the heading “Fab occupancy analysis.” No statistical measures were applied.

### Data and Software Availability

3D EM reconstructions have been deposited in the Electron Microscopy Databank (http://www.emdatabank.org/) under the accession numbers listed in the Key Resources Table. The crystallographic structure of the 10A Fab has been deposited in the Protein Data Bank (http://www.rcsb.org/) under accession number PDB: 6CJK.

## Supporting Citations

The following reference appears in the Supplemental Information: Chen et al. (2010).

## Supplemental Information

Document S1. Figures S1–S3 and Tables S1 and S2

Document S2. Article plus Supplemental Information
