# Supplementary material for: Electron-Microscopy-Based Epitope Mapping Defines Specificities of Polyclonal Antibodies Elicited during HIV-1 BG505 Envelope Trimer Immunization
Source: Immunity. 2018 Aug 21;49(2):288–300.e8. doi: 10.1016/j.immuni.2018.07.009 (PMC6104742; doi:10.1016/j.immuni.2018.07.009)
Supplement: Document S1. Figures S1–S3 and Tables S1 and S2 [file mmc1.pdf]

Immunity, Volume *49*

## Supplemental Information

# Electron-Microscopy-Based Epitope Mapping Defines Specificities of Polyclonal Antibodies Elicited during HIV-1 BG505 Envelope Trimer Immunization

**Matteo Bianchi, Hannah L. Turner, Bartek Nogal, Christopher A. Cottrell, David Oyen, Matthias Pauthner, Raiza Bastidas, Rebecca Nedellec, Laura E. McCoy, Ian A. Wilson, Dennis R. Burton, Andrew B. Ward, and Lars Hangartner**

# Figure S1

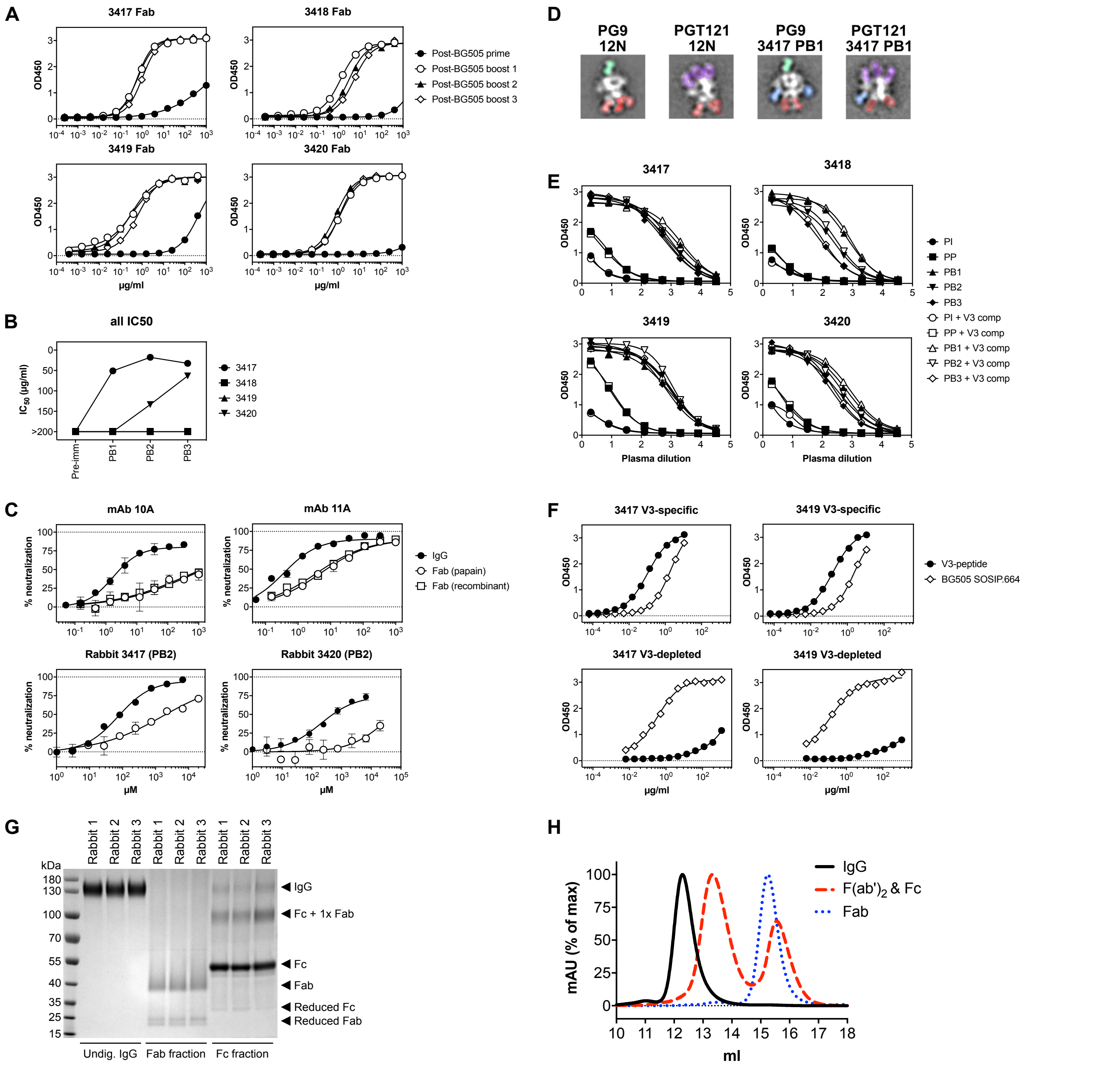


**Figure S1. Related to Figures 1, 2, and 3. ELISA and autologous neutralizing titers of polyclonal Fabs prepared from rabbits 3417-3420. (A)** Recombinant biotinylated BG505 SOSIP.664 was captured on neutravidin-coated ELISA plates before serial dilutions of rabbit Fabs were added. Fab binding was measured with an HRP-conjugated secondary anti-rabbit F(ab')2-specific antibody. **(B)** Autologous neutralizing antibody titers in purified IgG isolated from immunized rabbits. Serially diluted IgGs were first incubated with BG505 N332 pseudovirus and then infection assessed on TZM-bl cells by luciferase measurement. **(C)** Impact of enzymatic digestion and valency on neutralizing activity against BG505 N332. (Upper panels) Fabs of mAb 10A and 11A were either generated by digestion with papain or by recombinant expression in 293F cells. The neutralizing activity of the purified Fabs was then compared to the corresponding activity of the full IgG molecule as described above. (Lower panels) As above but using purified serum IgG or papain-digested Fab fragments prepared from serum of the indicated rabbits at PB2. **(D)** 2D class averages of complexes formed between the indicated mixes of mAb PG9, PGT121 and 12N, as well as with polyclonal Fabs from 3417 PB1. Fab densities are highlighted in false colors: blue for GH1, red for BOT, green for PG9 and purple for PGT121. **(E)** BG505 V3-competition ELISA. Performed as described above but incubating the rabbit plasma dilutions with 150$\mu$g/ml BG505 V3-peptide before adding them to the BG505 SOSIP.664-coated ELISA plates. **(F)** Detection of V3-specific Fabs in BG505 SOSIP.664-immunized rabbits. V3-specific Fabs were affinity-purified using biotinylated BG505 V3-peptide immobilized on streptavidin-agarose. Binding of the eluted Fabs (top panels) and the corresponding V3-depleted Fabs (bottom panels) to the V3-peptide or BG505 SOSIP.664 trimers was evaluated by ELISA as described above. **(G)** Unreduced SDS-PAGE showing purified serum IgGs, Fabs, and Fc fractions from 3 different control rabbits. IgGs were digested with Papain for 5-6h as described, and then Fabs purified (Fab fraction) by Protein A depletion of undigested/partially digested IgGs and Fc (Fc fraction). The faint bands showing partially reduced Fabs and Fc are due to residual cysteine present from the Fab digestion buffer. **(H)** SEC elution profile of purified Fabs (blue dotted line), compared to undigested IgGs (black line) and F(ab')2 + Fc resulting from IdeS digestion (red dashed line). The samples were run on a Superdex 200 increase 10/300 column. Note that Fabs run bigger than Fc on SEC, but smaller on SDS-PAGE.

# Figure S2

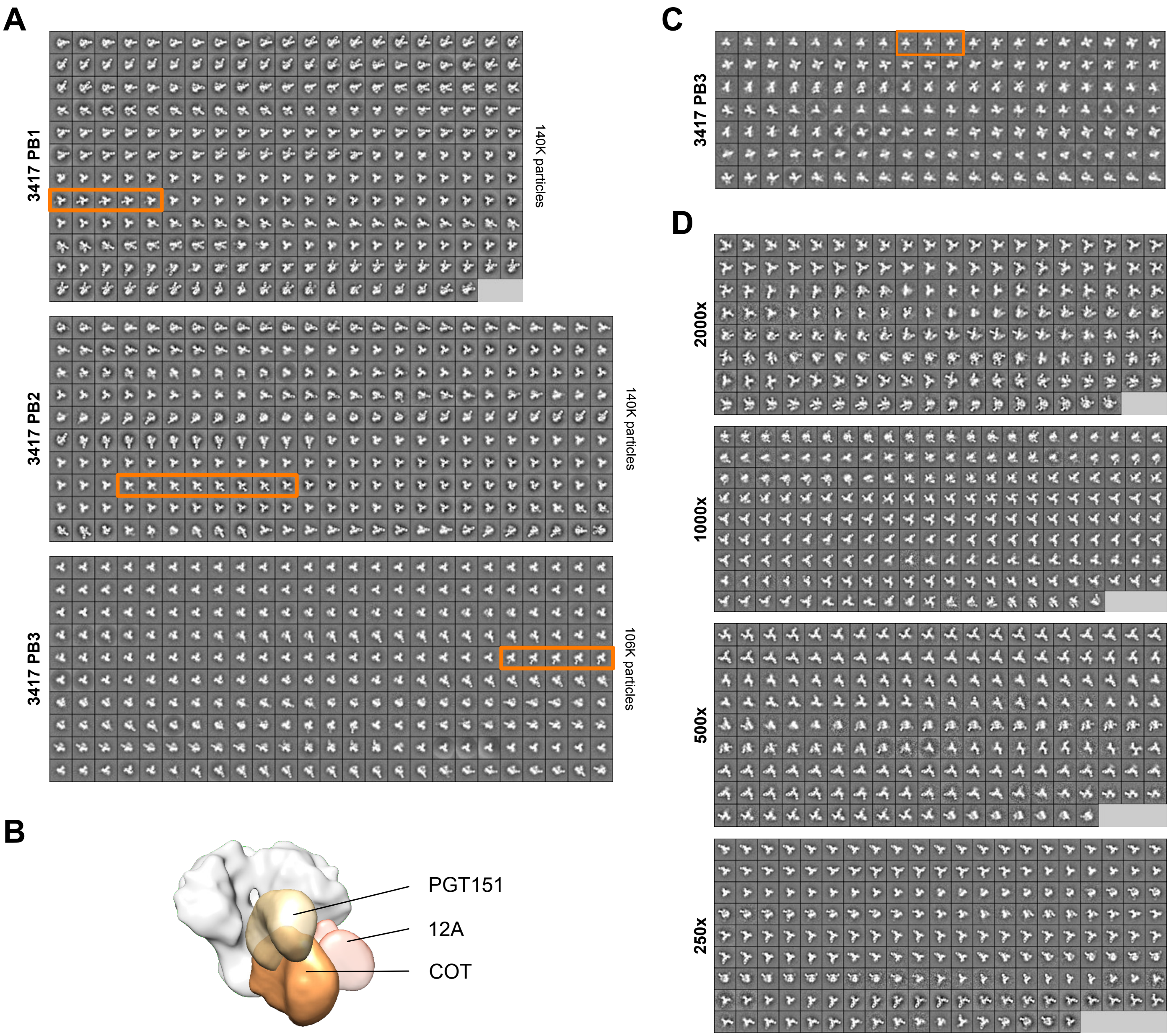


**Figure S2. Related to Figures 1 and 4. Impact of epitope silencing or Fab titration on antibody class detection. (A)** Increased detection of COT antibodies in immune complexes formed with a variant of BG505 SOSIP.664 in which the GH epitope was eliminated by the addition of N-linked glycosylation sites at positions 241 and 289 of gp120. 2D class averages with clearly visible COT antibodies are highlighted with orange boxes. The number of total particles used for 2D class averaging is indicated on the right. **(B)** Comparison of nsEM densities of antibodies binding to the fusion peptide region. Densities of COT-class Fabs, and Fabs of monoclonal antibodies PGT151 (EMDB 5918) and 12A are depicted, as indicated. **(C)** Increased detection of COT-class antibodies when both the glycan-hole and the BOT epitopes are silenced. Reference-free 2D class average of immune complexes formed between BG505 MD39 CPG9 and 3417 PB3 Fabs. The number of total particles used for 2D class averaging is indicated on the right, 2D-class averages showing occupancy of 3 COT Fabs are boxed. **(D)** Immune complexes formed with titrated amounts of 3417 PB2 Fab. Two-fold titrated amounts of 3417 PB2 Fab fragments, starting at 2000x $EC_{50}$ concentration, and a constant amount of BG505 SOSIP.664 was used for complex formation and nsEM. Reference-free 2D class averages are depicted with the number of particles averaged indicated on the right side.

# Figure S3

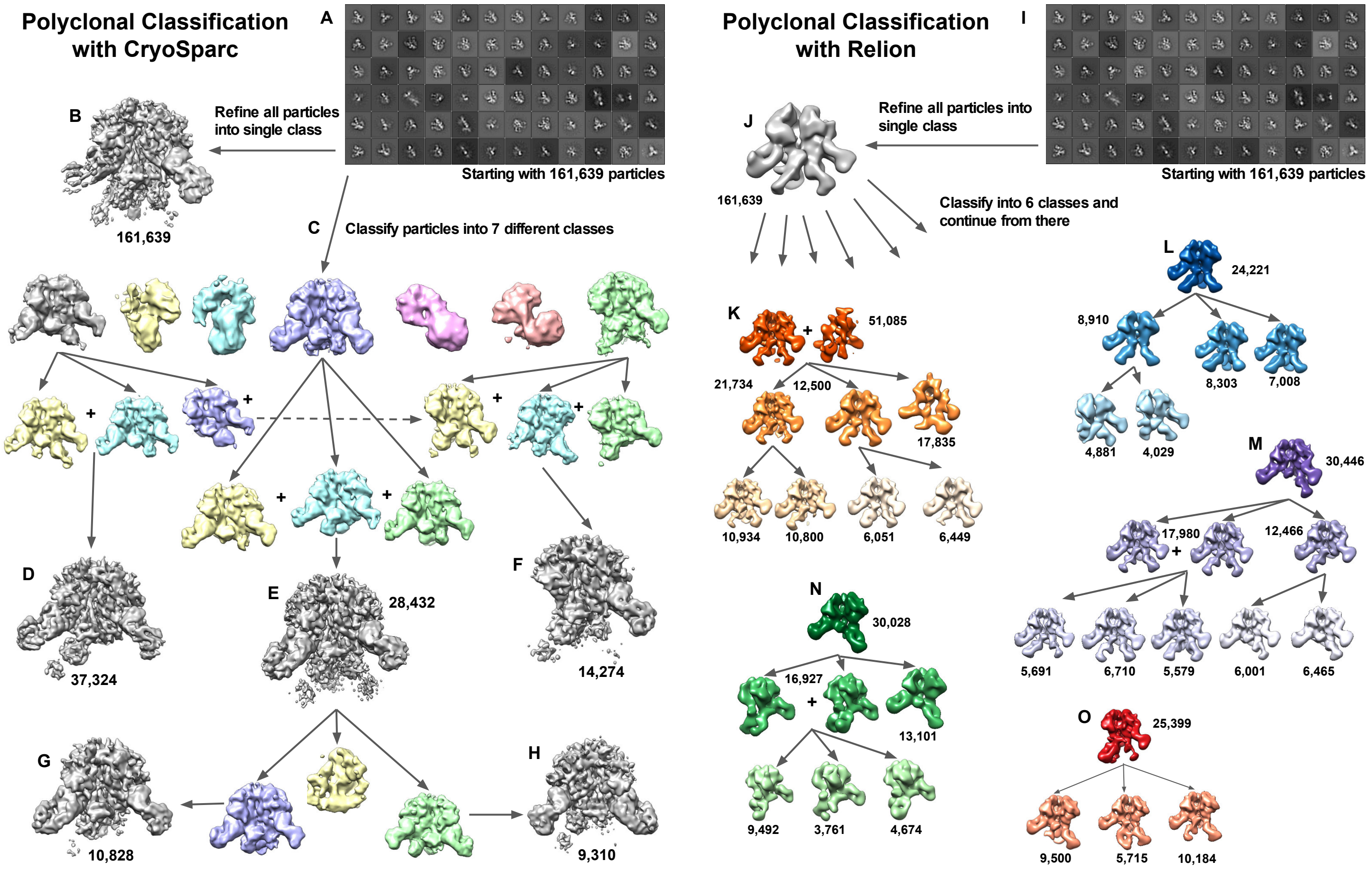


**Figure S3. Related to Figures 5 and 6. Generation and refinement of 3D models from cryoEM using CryoSparc (left) and Relion (right) software. (A)** CryoSparc 2D classification. All particles moved to CryoSparc for processing. **(B)** Ab-initio refinement of all particles into a single map. **(C)** All particles classified into 7 classes to separate occupancy differences and remove junk particles. **(D)** Refined map after a second round of classification and removing a class shown to have only one glycan hole (GH) Fab bound. **(E)** Refined class after two subsequent classifications showed no significant differences in 3D maps. **(F)** Refined map after adding particles from a separate class shown to have a single GH Fab bound. **(G)** and **(H)** Final map (E) showed substoichiometric density for bottom binders and had enough particles for more 3D classification. **(I)** Same particles also processed in Relion. **(J)** All particles refined into a single map using unliganded trimer as starting model. Performed 3D classification into 6 classes using (J) as initial model. **(K)** Particles from two original classes were combined and classified into three classes to separate substoichiometric antibody binding and remove junk. **(L)** Single class was classified into three more classes. Two classes showed complete Fab density and classification stopped there. Third class was classified into two more classes. **(M)** Single class was classified into three more maps. Particles with similar Fab density were combined and further classified. **(N)** Single class was 3D classified into three more maps. One map showed complete Fab density. Others were combined and classified into three more classes. **(O)** Map was 3D classified into three more classes, some showing full Fab density while others still had substoichiometric occupancy. With fewer particles, classification ended there.

## Table S1

| Complex composition | Calculated mAb occupancy | Elution volume |
|---|---|---|
| No antibody (BG505 SOSIP.664 only) | 0 | 14.33 |
| PGT145 | 1 | 14.25 |
| PG9 | 1 | 14.21 |
| PG16 | 1 | 14.27 |
| VRC01 | 2 | 14.00 |
| PGV04 | 2 | 14.10 |
| *PGT151** | *2* | *14.50* |
| 3BC315 | 2 | 14.20 |
| 12A | 2 | 14.00 |
| PGT121 | 3 | 13.63 |
| *35O22** | *3* | *14.54* |
| 12N | 3 | 13.61 |
| 11A | 3 | 13.71 |
| 10A | 3 | 13.67 |
| 10-1074 | 3 | 13.60 |
| PG9+VRC01 | 3 | 13.68 |
| PG16+10A | 4 | 13.43 |
| PGT145+PGT121 | 4 | 13.51 |
| VRC01+12A | 4 | 13.63 |
| VRC01+3BC315 | 4 | 13.72 |
| VRC01+12A | 4 | 13.65 |
| 12N+PG9 | 4 | 13.35 |
| PG9+PGT121 | 4 | 13.51 |
| *PGT121+PGT151** | *5* | *13.54* |
| PGT121+VRC01 | 5 | 13.35 |
| PGT145+VRC01+12A | 5 | 13.46 |
| PG9+PGV04+3BC315 | 5 | 13.58 |
| *12N+PGT151** | *5* | *13.65* |
| 12N+12A | 5 | 13.50 |
| 12N+VRC01 | 5 | 13.23 |
| VRC01+10A | 5 | 13.28 |
| 10-1074+10A | 6 | 12.91 |
| PG16+VRC01+10A | 6 | 13.17 |
| 12N+PGT121 | 6 | 12.92 |
| 12N+10A | 6 | 13.26 |
| *PGT121+VRC01+PGT151** | *7* | *13.36* |
| PGT121+VRC01+12A | 7 | 13.14 |
| 10-1074+PGV04+12A | 7 | 13.19 |
| PGT121+PGV04+3BC315 | 7 | 13.29 |
| 10-1074+PGV04+3BC315 | 7 | 13.25 |
| 12N+PG9+PGT121 | 7 | 12.85 |
| 12N+PG9+10A | 7 | 13.04 |
| 12N+PGT145+PGT121 | 7 | 12.85 |
| PGT121+VRC01+12A | 7 | 13.13 |
| PGT121+VRC01+10A | 8 | 12.84 |
| *12N+PGT121+PGT151** | *8* | *12.93* |
| 12N+PGT121+VRC01 | 8 | 12.75 |
| 12N+VRC01+10A | 8 | 13.02 |
| 12N+PGT121+10A | 9 | 12.71 |
| 12N+PG9+PGT121+VRC01 | 9 | 12.70 |
| 12N+PG9+PGT121+10A | 10 | 12.61 |
| 12N+PGT145+PGT121+10A | 10 | 12.65 |
| 12N+PGT121+VRC01+10A | 11 | 12.56 |
| 12N+PG9+PGT121+VRC01+10A | 12 | 12.53 |
| 12N+PGT145+PGT121+VRC01+10A | 12 | 12.58 |

**Table S1. Related to Figure 3. Calculated mAb occupancy and elution volumes for immune complexes formed with saturating concentrations of the indicated mAbs.** Complexes containing 35O22 and PGT151 (italicized) were excluded from the analysis due to abnormal behavior on SEC. Fab 35O22 displayed a considerably prolonged retention in SEC (probably due to stickiness to the matrix). Excessive proteolytic degradation did not account for this observation as the Fab displayed the expected molecular weight in SDS PAGE (not shown). Thus, it is likely that 35O22 non-specifically interacts with the SEC matrix. Moreover, complexes only containing 35O22 or PGT151 Fabs ran at a molecular weight even smaller than uncomplexed BG505 SOSIP.664.

## Table S2

| Data collection | Fab 10A |
|---|---|
| Beamline | APS 23ID-D |
| Wavelength (Å) | 1.03315 |
| Space group | P1 |
| Unit cell parameters(Å, °) | a=51.17, b=63.56, c=72.65, α=67.99, β=87.87, γ=76.74 |
| Resolution (Å) | 40.7-1.80 (1.83-1.80)[a] |
| Unique reflections | 73,928 (3,385)[a] |
| Redundancy | 3.2 (2.6)[a] |
| Completeness (%) | 96.3 (87.8)[a] |
| $<I/\sigma_I>$ | 16.5 (1.0)[a] |
| $R_{sym}$[b] (%) | 7.4 (93.8)[a] |
| $R_{pim}$[b] (%) | 4.8 (66.1)[a] |
| $CC_{1/2}$[c] (%) | 85.6 (32.9)[a] |
| **Refinement statistics** | |
| Reflections (work) | 70,074 |
| Reflections (test) | 3,841 |
| $R_{cryst}$[d] / $R_{free}$[e] (%) | 16.3/21.0 |
| No. of atoms | |
| Protein | 6,415 |
| Water | 547 |
| Ligands | 18 |
| Average *B*-value (Å$^2$) | |
| Protein | 36 |
| Water | 41 |
| Ligands | 48 |
| Wilson *B*-value (Å$^2$) | 27 |
| **RMSD from ideal geometry** | |
| Bond length (Å) | 0.011 |
| Bond angle (°) | 1.07 |
| **Ramachandran statistics (%)**[f] | |
| Favored | 97.15 |
| Outliers | 0.12 |

[a] Numbers in parentheses refer to the highest resolution shell.

[b] $R_{sym} = \Sigma_{hkl} \Sigma_i | I_{hkl,i} - <I_{hkl}> | / \Sigma_{hkl} \Sigma_i I_{hkl,i}$ and $R_{pim} = \Sigma_{hkl} (1/(n-1))^{1/2} \Sigma_i | I_{hkl,i} - <I_{hkl}> | / \Sigma_{hkl} \Sigma_i I_{hkl,i}$, where $I_{hkl,i}$ is the scaled intensity of the i$^{th}$ measurement of reflection h, k, l, $<I_{hkl}>$ is the average intensity for that reflection, and *n* is the redundancy.

[c] $CC_{1/2}$ = Pearson correlation coefficient between two random half datasets.

[d] $R_{cryst} = \Sigma_{hkl} | F_o - F_c | / \Sigma_{hkl} | F_o | \times 100$, where $F_o$ and $F_c$ are the observed and calculated structure factors, respectively.

[e] $R_{free}$ was calculated as for $R_{cryst}$, but on a test set comprising 5% of the data excluded from refinement.

[f]Calculated from MolProbity (Chen et al., 2010)

**Table S2. Related to Figure 5. X-ray data collection and refinement statistics for Fab 10A (PDB 6CJK).**
